# Towards a map of the immune system manipulation network by *Trypanosoma cruzi*

**DOI:** 10.3389/fcimb.2025.1711520

**Published:** 2026-01-06

**Authors:** Juan Cruz Gamba, Ana Rosa Pérez, Carolina Verónica Poncini, Cristina Poveda, Iván Marcipar, Gabriel Cabrera

**Affiliations:** 1 Facultad de Bioquímica y Ciencias Biológicas, Universidad Nacional del Litoral, Santa Fe, Argentina; 2 Instituto de Investigación en Señales, Sistemas e Inteligencia Computacional (CONICET‐UNL), Facultad de Ingeniería y Ciencias Hídricas, Santa Fe, Argentina; 3 Instituto de Inmunología Clínica y Experimental de Rosario (IDICER-CONICET), and Facultad de Ciencias Médicas, Universidad Nacional de Rosario, Santa Fe, Argentina; 4 Centro de Investigación y Producción de Reactivos Biológicos (CIPReB), Facultad de Ciencias Médicas, Universidad Nacional de Rosario, Santa Fe, Argentina; 5 Laboratorio de Inmunología Celular e Inmunopatología de Infecciones, IMPaM UBA-CONICET, Departamento de Microbiología, Parasitología e Inmunología, Facultad de Medicina, Universidad de Buenos Aires, Ciudad Autónoma de Buenos Aires, Buenos Aires, Argentina; 6 Department of Pediatrics, National School of Tropical Medicine, Baylor College of Medicine, Houston, TX, United States

**Keywords:** Chagas disease, evasion, immune system, manipulation, subversion, *Trypanosoma cruzi*

## Abstract

*Trypanosoma cruzi* (*T. cruzi*), the protozoan parasite that causes Chagas disease, remains a major public health challenge, with more than six million people infected worldwide. Despite more than a century of research and extensive evaluation of different strategies, no vaccine has progressed to late-phase clinical trials. This failure highlights the need to better understand host–parasite interactions, with special emphasis on the immunoregulatory pathways exploited by the parasite. In this review, we propose an initial comprehensive map of the *T. cruzi* immune manipulation network, integrating research on numerous parasite and host components involved. Five main cores of manipulation are proposed, including how *T. cruzi* skews macrophage polarization toward regulatory profiles, the impairment of dendritic cell maturation and Th1 induction, resistance to and subversion of complement pathways, expansion of myeloid-derived suppressor cells (MDSCs), and suppression and delay of adaptive immunity by driving non-specific B-cell activation, thymic atrophy, and T-cell dysfunction. Mapping these mechanisms may reveal how parasite molecules such as trans-sialidases, cruzipain, proline racemase, mucin-associated surface proteins, complement regulatory proteins, and others interact in a complex network of manipulated immune pathways. A deeper understanding of these interactions could have significant implications for immunotherapeutic strategies. Future vaccine designs may benefit from rationally selected combinations that maximize targeted effector responses while minimizing the manipulation of the immune network by *T. cruzi*.

## 1 Introduction

Despite the success of many vaccines at controlling disease and reducing morbidity, a long list of pathogens still lack effective vaccines (Plotkin, 2018). Several of these resistant microorganisms share some features such as: genetic variability, a complex life cycle, and the ability to evade and subvert the host immune system. *Trypanosoma cruzi* (*T. cruzi*), the etiological agent of Chagas disease (CD), is a protozoan parasite that exhibits all these characteristics (Cardillo et al., 2015; Cardoso et al., 2015; Geiger et al., 2016a; Morrot et al., 2016; Cabrera and Marcipar, 2019; Farani et al., 2024).

*T. cruzi* alternates between a mammalian vertebrate host and an invertebrate hematophagous vector, which is an insect belonging to the subfamily Triatominae (order Hemiptera, family Reduviidae) (Acevedo et al., 2018). The parasite uses different forms in its complex life cycle, including trypomastigotes, epimastigotes, and amastigotes. The trypomastigote is an infective and nondividing form of the parasite. Additionally, this form can be subdivided into metacyclic trypomastigotes (mT), which are the forms that initially infect mammals, and blood trypomastigotes (bT), which are infective forms released into the blood from infected cells and can invade other cells or infect a vector after feeding. The epimastigote is a form that can replicate in the midgut of the insect vector. The amastigote is an intracellular form that can divide inside vertebrate host cells. Alternatively, it was reported that if amastigotes are released during the lysis of a cell, they can also display infective capacity (Acevedo et al., 2018; Lidani et al., 2019). More recently, using transmission electron microscopy and scanning electron microscopy, more detail regarding these initial forms is being acquired. In this sense, the passage from epimastigotes to mT has been divided into three more forms, and the passage from amastigotes to bT has been shown to include at least three more intermediates, including a transitional or epimastigote-like form (Tomasina et al., 2024).

The most common routes of transmission to humans are vectorial, blood transfusion, congenital, and oral (Acevedo et al., 2018). The majority of the patients infected via the vectorial route pass through an asymptomatic acute phase, only displaying, in some cases, fever, hepatosplenomegaly and inflammatory reactions. After a period of nearly 2 to 4 months, the individuals begin a chronic phase that may end after 10–20 years with the development of symptoms such as irreversible damage to the heart, esophagus, and colon in nearly a third of the patients (MaChado et al., 2012; Barrias et al., 2013). Currently, more than 6 million people are estimated to be infected with *T. cruzi* in endemic and non-endemic countries, and more than 60 million are living in areas at risk of infection (Morillo et al., 2017; Sales Junior et al., 2017; Lidani et al., 2019; Farani et al., 2024; Teixeira et al., 2025).

Basic principles of the parasite’s life cycle and host immune response have already been thoroughly described (Ley et al., 1988; Gazzinelli et al., 1991; Oliveira et al., 2004, 2010; Padilla et al., 2009; Gravina et al., 2013; Lidani et al., 2017; Acevedo et al., 2018). However, a summary is provided in Supplementary Table I to contextualize subsequent discussions. Almost all types of classical vaccines have been assessed, including live attenuated, subunit, and recombinant vaccines (Dumonteil et al., 2012; Arce-Fonseca et al., 2015; Rodríguez-Morales et al., 2015; Sánchez-Valdéz et al., 2015; Dumonteil and Herrera, 2021; Farani et al., 2024; Teixeira et al., 2025).

**Figure 1:**
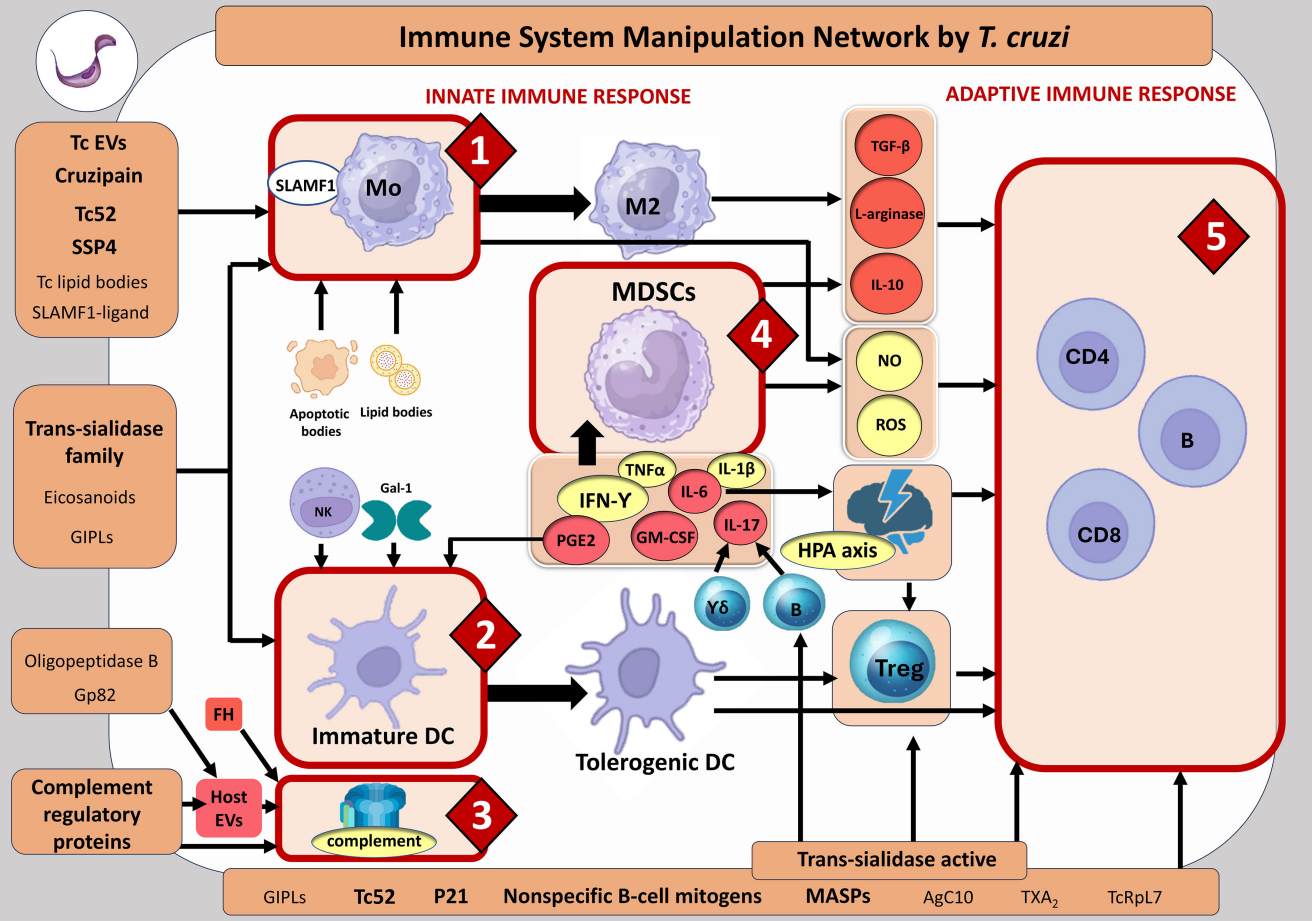
Subversion network of the immune system by *T. cruzi.* Parasite components that have been assessed as vaccine candidates are marked in bold. INNATE IMMUNE RESPONSE. Core 1: Manipulating macrophage polarization. *T. cruzi* elements involved: Tc EVs: *T. cruzi* extracellular vesicles. They can promote immunoregulatory macrophage polarization. SlamF1-ligand (Signaling lymphocytic activation molecule family member 1-ligand): Indirectly dampens effector immunity. Cruzipain: A major cysteine protease that has been implicated in several pathways of host immune manipulation, including macrophage polarization. SSP4 (amastigote-specific surface protein): Causes T-cell immunosuppression by stimulating NO production by macrophages, which in turn may suppress T-cell responses. Tc52: A glutathione disulfide thioltransferase that may play a dual role affecting both effector and immunoregulatory pathways of the innate and adaptive immune system. Tc lipid bodies: May contribute to impairing macrophage Th1 polarization. Trans-sialidase family: A large group of proteins with multiple subgroups that contribute to diverse mechanisms of immune evasion and subversion. Eicosanoids: Thromboxane A_2_ (TXA_2_) and Prostaglandin E_2_ (PGE_2_) participate in immunoregulatory pathways that affect both macrophages and dendritic cells. GIPLs (glycoinositolphospholipids): Play a dual role, activating a proinflammatory response, but also modulating macrophage and DC functions. Additionally, they can block CD4+ and CD8+ T-cell proliferation. Host components subverted: Apoptotic bodies: Uptake of apoptotic cells can favor Th2-type polarization of macrophages. Host lipid bodies: Participate in PGE_2_ production and Th2 polarization. Host PGE_2_: Together with TGF-β, may favor Th2 polarization. Core 2: Avoiding the triggering of a Th1 response by dendritic cells. *T. cruzi* elements involved: Trans-sialidase family: *T. cruzi* can modulate DC function via sialylated mucins and Siglec receptors. Eicosanoids: Thromboxane A_2_ (TXA_2_) and Prostaglandin E_2_ (PGE_2_) affect DC effector functions. GIPLs: Reported to interfere with DC responses. Host components subverted: NKs: IL-10-producing NKs can control survival of immature DCs. Gal1: This galectin can drive immunoregulatory T-cell responses by affecting DCs. Core 3. Resisting and subverting the host complement system. *T. cruzi* elements involved: Oligopeptidase B and gp82: Involved in processes that increase EVs, contributing to complement evasion by the parasite. Complement regulatory proteins: Several proteins such as calreticulin, Tc-DAF, TcCRP, TcCRIT, and gp58/68 participate in pathways related to complement evasion. Host components involved: EVs: These vesicles carry host complement receptors like CR1 and DAF and can contribute to inhibiting C3 convertase activity. FH: A host complement regulatory protein that can be used by the parasite to evade complement activation. Core 4: Subversion of MDSCs. Host components involved: Cytokines: Infection affects the levels of IFN-γ, IL-6, TNF-α, PGE_2_, IL-17, and GM-CSF, all molecules that have been involved in MDSC induction. γδ T cells: IL-17 production by these cells has been related to MDSC induction. B cells: They can be induced to produce IL-17 by active TS, an event that may also lead to MDSC increases. ADAPTIVE IMMUNE RESPONSE. Core 5: Suppressing and delaying the adaptive immune response. *T. cruzi* elements involved: P21: May be involved in latency and host immune response evasion. Nonspecific B-cell mitogens: Several proteins such as proline racemase, Tc24, and the shed acute phase antigen (SAPA) have been shown to promote the production of antibodies not directed against the parasite. MASPs (mucin-associated surface proteins): These polymorphic proteins have been proposed to favor immune system escape. AgC10: A mucin that can inhibit T-cell proliferation and block IL-2 synthesis. TXA_2_: Thromboxane A_2_ from *T. cruzi* has been related to thymocyte apoptosis. TcRpL7: A repetitive fragment of the ribosomal protein L7a that may suppress B-cell proliferation. Trans-sialidase family: Contributes to thymus atrophy and impairs T effector cell function. Host components involved: IL-10: An anti-inflammatory cytokine that can inhibit CD4+ T-cell proliferation. L-arginase: Depletes L-arginine, suppressing T-cell activation. TGF-β: Inhibits cytotoxic T lymphocytes and Th1- and Th2-cell differentiation, favoring Tregs and tolerance. ROS: Can affect neighboring T cells, impairing proliferation and effector functions. NO: High levels of NO can induce T-cell apoptosis. It can react with superoxide to form peroxynitrites, which inhibit T-cell proliferation and effector functions. HPA axis: Affected by infection and can alter the balance of the broad immune response, influencing thymocyte maturation and thymus cell output. Tregs: They can suppress the effector immune response. Image created using www.biorender.com.

The setbacks in advancing to late-phase clinical trials suggest that a better understanding of host–parasite interactions may be necessary to develop new strategies that complement classical vaccination approaches by analyzing the scenario from additional perspectives. To date, the need for innovative approaches to develop successful vaccines has also been suggested for several complex pathogens such as *Mycobacterium tuberculosis*, HIV, or *Plasmodium falciparum* (Moise et al., 2014; Ndure and Flanagan, 2014; Li et al., 2015; Wilson et al., 2015; Schaible et al., 2017; Batista-Duharte et al., 2018; Dorhoi et al., 2020; Duffy and Patrick Gorres, 2020; Prochetto et al., 2022). Bearing this in mind, this review aims to outline a *T. cruzi* manipulation network that integrates the diverse pathways employed by the parasite to evade and subvert the host immune system. To this end, five central manipulation cores are proposed, described below, and illustrated in **Figure 1**. In addition, Supplementary Table II lists the parasite components depicted in the figure that have been reported to play key roles in immune modulation and may therefore represent potential targets for chemotherapeutic or vaccine approaches.

It should be noted that, since this is an attempt to integrate information from various *in vitro* and *in vivo* studies using distinct models, special care must be taken when considering differences in parasite strains, host genetic background, route of infection, parasite inoculum, and other variables that may influence the pathways described in this general scenario.

## 2 Core 1. Manipulating macrophage polarization

### 2.1 Introduction

*T. cruzi* is capable of infecting any nucleated cell through active or passive mechanisms (Cardoso et al., 2015). This section focuses on the interaction of the parasite and macrophages, as these immune cells are widely distributed in tissues and are among the first to be infected by *T. cruzi* (Romano et al., 2012; Cardoso et al., 2015; Acevedo et al., 2018). Macrophages can both kill the parasite and elicit immune responses (Muñoz-Fernández et al., 1992) or allow parasite multiplication (Goes et al., 2016; Paiva et al., 2018), making this early interaction critical for pathogen persistence or eradication.

In general terms, stimulated macrophages can be polarized to M1, which are associated with a Th1-type response, or to M2, which are associated with a Th2-type response. Since a Th1-type response implies the production of IL-12 and IFN-γ, which are detrimental to the parasite, *T. cruzi* has evolved numerous strategies to infect and favor M2 polarization, which is beneficial for its persistence through the production of IL-10 and TGF-β (Cerbán et al., 2020).

### 2.2 Cell infection

The invasive trypomastigote forms of *T. cruzi* are highly motile and slender parasites (~20 µm long; 2 µm wide), capable of infecting macrophages through at least three active entry pathways or passively by subverting phagocytosis (Caradonna and Burleigh, 2011; Walker et al., 2014). Regardless of the mechanism employed, all pathways lead the parasite to lysosomal-based endosomes (Andrade and Andrews, 2005; Walker et al., 2014; Teixeira et al., 2023).

At least the following components of the parasite were described to be involved in macrophage infection by the active pathways: proteins of the trans-sialidase (TS) superfamily (Flávia Nardy et al., 2015; Horta et al., 2020), cruzipain (Scharfstein et al., 2000), Ecto-ATPases (Santos et al., 2009), Ecto-tyrosine phosphatase (Gallo et al., 2011), Trypomastigote Small Surface Antigen (TSSA) (Cámara et al., 2017), oligopeptidase B (Horta et al., 2020), Tc- phspholipase A1 (Tc-PLA1), Signaling Lymphocytic Activation Molecule Family member 1-ligand (SLAMF1-ligand) (Calderón et al., 2012; Poveda et al., 2020; Herreros-Cabello et al., 2024), and extracellular vesicles (EVs) (de Pablos Torró et al., 2018).

The best-characterized proteins involved in adhesion belong to group II of TS superfamily and are expressed by mammalian-infective stages of *T. cruzi*, including bloodstream and tissue culture-derived trypomastigotes, metacyclic trypomastigotes, and amastigotes (Freitas et al., 2011).

*T. cruzi* invasion also seems to depend on enzymes whose active sites face the external medium rather than the cytoplasm (called ecto-enzymes). Ecto-ATPases hydrolyze extracellular nucleoside tri-and/or diphosphates such as ATP, and *T. cruzi* ecto-enzyme inhibition was shown to inhibit, to a certain extent, macrophage infection (Santos et al., 2009; Gallo et al., 2011).

TSSA is a highly antigenic, GPI-anchored protein on the trypomastigote coat whose adhesive properties rely on exposed peptide motifs that may mediate host cell receptor interaction before internalization (Cámara et al., 2017).

Active pathways of *T. cruzi* infection require transient and localized Ca^2+^ increases. Two parasite enzymes have been implicated in this process: oligopeptidase B and cruzipain.

Oligopeptidase B is a cytosolic serine endopeptidase that generates a product recognized by a G-protein-coupled receptor involved in Ca^2+^ increases (Burleigh et al., 1997). In addition, the cysteine protease cruzipain cleaves host kininogen into bradykinin, which interacts with its classical bradykinin receptor on the host, eliciting Ca^2+^ increases (Scharfstein et al., 2000; Horta et al., 2020). Cruzipain may also activate latent TGF-β, favoring macrophage infection (Ferrão et al., 2015).

Tc-PLA1 has been proposed as an important *T. cruzi* virulence factor involved in cell invasion (Belaunzarán et al., 2013).

*T. cruzi* also exploits host immune receptors to facilitate entry. The receptor SLAMF1 (CD150) influences infection in a strain-dependent manner by modulating parasite entry and macrophage oxidative responses. Studies using representatives of the six *T. cruzi* DTUs showed that SLAMF1-deficient macrophages generally had lower parasite loads, increased NOX2 expression, and higher reactive oxygen species (ROS) production compared to wild-type BALB/c controls, except for the VFRA strain, which showed the opposite pattern. This indicates that *T. cruzi* can manipulate SLAMF1-mediated ROS regulation to favor replication, with effects varying by parasite genotype (Poveda et al., 2020).

Another potential mechanism that may be involved in macrophage infection is the release of *T. cruzi* extracellular vesicles (EVs), which are enriched in glycoproteins of the TS superfamily and α-galactosyl-containing glycoconjugates (Trocoli Torrecilhas et al., 2009; Lovo-Martins et al., 2018). EVs shed by *T. cruzi* might act on macrophages by increasing parasite adherence or through acid phosphatase activities (Neves et al., 2014).

Extracellular vesicles (EVs) are particles released by cells, delimited by a lipid bilayer, and incapable of self-replication. According to the International Society for Extracellular Vesicles (ISEV), EVs can be classified into small EVs (<200 nm in diameter) and large EVs (>200 nm in diameter) (Welsh et al., 2024). Since most studies in the field do not discriminate between vesicle sizes, the general term *EVs* will be used throughout this work, except for apoptotic bodies, which range from 500 to 4000 nm and have been distinguished in the original studies based on their origin (de Pablos Torró et al., 2018).

Numerous studies have addressed the protein and RNA composition of epimastigote or trypomastigote-derived *T. cruzi* EVs, their potential use as vaccine or diagnostic tools, and the effects of these vesicles on parasite infectivity and on components of the immune system (Garcez et al., 2023). In line with this topic, only studies related to immune system subversion during the acute phase of trypomastigote infection are described.

Accumulated evidence supports that EVs carry virulence factors that, after endocytosis, are released into the cytoplasm, favoring parasite infection by affecting cell polarization, membrane permeability, intracellular calcium concentration, cytoskeletal integrity, apoptosis, inhibition of C3 convertase, and other processes (Cestari et al., 2012; Bayer-Santos et al., 2013; Retana Moreira et al., 2019; Cornet-Gomez et al., 2023b; Ansa-Addo et al., 2024). It is important to note that EVs from different parasite strains release distinct cargoes and can therefore elicit different effects on immune cells (Nogueira et al., 2015; Lovo-Martins et al., 2018; Ribeiro et al., 2018; Meneghetti et al., 2025).

Most studies agree that, despite differences in the cargo associated with *T. cruzi* strains, EVs enhance the infective capacity of the parasites, mainly by pathways that modulate or decrease the inflammatory response (Lovo-Martins et al., 2018; D’Avila et al., 2021; Dos Santos et al., 2023), but also by using pathways that do not decrease the inflammatory response (Choudhuri and Garg, 2020; Cronemberger-Andrade et al., 2020).

Other molecules implicated in host attachment and invasion include members of the mucins and mucin-associated surface proteins (MASPs) superfamilies, as well as smaller protein families such as dispersed gene family-1 (DGF-1) proteins, penetrin (Herrera et al., 1994), and gp63s metalloproteases. While their precise roles remain incompletely understood, these molecules may act in synergy with the well-characterized surface factors to facilitate host-parasite interaction (Caradonna and Burleigh, 2011).

In addition to active pathways, passive invasion can occur when macrophages phagocytose the parasite. *T. cruzi* calreticulin (TcCRT) interaction with the C1 component of the complement generates an “*eat me*” signal that mimics a physiological apoptotic cell removal signal, because apoptotic cells translocate host CRT to the external membrane (Ramírez et al., 2011; Sosoniuk-Roche et al., 2017).

Finally, prostaglandin E2 (PGE2) secreted by *T. cruzi* lipid bodies, as well as by the host, could also play a role in macrophage infection, as aspirin pretreatment of peritoneal macrophages markedly inhibited *T. cruzi* invasion, while PGE2 addition restored the infection capacity (Malvezi et al., 2014; de Almeida et al., 2018).

### 2.3 Resistance and phagolysosome escape

Irrespective of the mechanism of infection, the first challenge for the parasite consists in managing the oxidative environment inside the phagolysosome. To counteract this, *T. cruzi* employs at least five peroxidases and four superoxide dismutases (SODs) (Piacenza et al., 2013; Cardoso et al., 2015). Although oxidative stress has traditionally been viewed as detrimental to the parasite, evidence suggests a more complex relationship, as it can also enhance *T. cruzi* infection: treatment of macrophages with ROS scavengers reduces parasite burden, whereas exposure to H_2_O_2_ promotes parasite replication (Goes et al., 2016; Paiva et al., 2018).

Then, for vacuolar escape, *T. cruzi* relies on its active trans-sialidase, which desialylates lysosome-associated membrane proteins 1 and 2 (LAMP-1 and LAMP-2) on the internal surface of the vacuoles. This event would be necessary for a putative vacuole-lysing hemolysin called Tc-TOX to generate pores, disrupting the phagolysosome (Albertti et al., 2010; Caradonna and Burleigh, 2011). Evidence further suggests that parasites may exploit oxidative stress even after reaching the cytoplasm, as amastigote growth was decreased when an antioxidant response was generated (Paiva et al., 2018).

Interestingly, there is evidence that *T. cruzi*, through its P21 protein, can regulate intracellular parasite multiplication to avoid excessive parasitemia, which may be detrimental to both the host and the parasite. Maintaining replication under control has been postulated as a mechanism that favors latency and immune evasion (da Silva et al., 2009; Teixeira et al., 2019; Silveira et al., 2024).

### 2.4 Manipulation

The profile of macrophages is closely linked to how they metabolize L-arginine. Th1-like macrophages use L-arginine to produce NO via inducible nitric oxide synthase (iNOS), supporting microbicidal activity. In contrast, Th2-like macrophages metabolize L-arginine through arginase-1, producing urea as a byproduct (Giordanengo et al., 2002; Cerbán et al., 2020).

As previously mentioned, *T. cruzi* actively works to suppress a Th1-response (Cerbán et al., 2020), which is detrimental not only for the parasite but also for the host, since exacerbated tissue damage and inflammation can compromise the life of both pathogen and host (Arocena et al., 2014). At least the following components have been involved in macrophage manipulation: cruzipain, amastigote-specific surface protein (SSP4), GIPLs, Tc52, lipid bodies, and extracellular vesicles (EVs). Additionally, it exploits host-derived factors, including host-lipid bodies, apoptotic bodies, and EVs, to skew macrophage polarization toward Th2 dominance (**Figure 1**).

In addition to its role in allowing host invasion, cruzipain has been broadly associated with a function related to redirecting macrophage polarization from Th1 to a Th2 profile. In this sense, it was shown that human rNF- κB p65 could be proteolytically cleaved by cruzipain very early during cell infection (Doyle et al., 2011). Moreover, targeting NF-κB p65 impaired mouse macrophage activation and IL-12 secretion, while allowing parasite survival and increasing arginase-1 expression. Supporting the role of cruzipain in NF-κB p65 inactivation, mouse macrophages infected with cruzipain-deficient parasites rapidly activated, showing NF-κB activation, IL-12 secretion, and parasiticidal activity. Moreover, pre-activation of macrophages with LPS was able to kill *T. cruzi* parasites, but LPS stimulation post-infection fails to clear the parasite, suggesting that *T. cruzi* establish irreversible control of the host cells (Doyle et al., 2011).

In addition, it was also reported that cruzipain may favor an alternative activation of macrophages, which includes an increase in arginase-1 activity and higher levels of IL-10 and TGF-β cytokines in culture supernatants (Giordanengo et al., 2002; Stempin et al., 2002; Cerbán et al., 2020). Further supporting a role of cruzipain in eliciting a Th2 profile, immunization with cruzipain caused the development of a splenic Th2-type cytokine (Giordanengo et al., 2002).

Despite the proinflammatory and activating role described for GIPLs (De Arruda Hinds et al., 1999; Almeida and Gazzinelli, 2001; Medeiros et al., 2007), it has also been described that preincubation of macrophages with GIPLs inhibits LPS-induced TNF-α, IL-12, and even IL-10 production, suggesting a strong influence of GIPLs on macrophages. In addition, GIPLs led to a down-regulation of CD80, CD86, CD54, CD40, and HLA-DR expression on the surface of LPS-stimulated macrophages (Brodskyn et al., 2002). Finally, it was also described that GIPLs were able to induce macrophage apoptosis in the presence of IFN-γ, which could lead to amastigote and trypomastigote release (DosReis et al., 2002).

Tc52 has been reported to act on macrophages in several ways, modulating the expression of IL-1α, IL-10, and IL-12 mRNA, and thus influencing Th1- or Th2-type immune polarization, and synergizing with IFN-γ to enhance nitric oxide production, an event that may also affect macrophage involvement during *T. cruzi* infection.

It was postulated that *T. cruzi* SSP4, which is released by phospholipase C, causes T-cell immunosuppression by stimulating NO production by macrophages (Ramos-Ligonio et al., 2004).

Lipid bodies (LBs) are lipid-rich organelles that have been found in almost all organisms from bacteria to humans. It was reported that PGE2 from *T. cruzi* LBs may contribute to impairing macrophage Th1 polarization (de Almeida et al., 2018).

*T. cruzi* EVs carry several virulence factors that can modulate macrophages *in vitro* in several ways, including the formation of host lipid bodies and the production of PGE_2_, leading to a more favorable environment for parasite infection, with an alteration of the balance between the pro-inflammatory and regulatory cytokines toward an immunoregulatory profile (de Pablos Torró et al., 2018; Lovo-Martins et al., 2018). *In vivo*, EV inoculation prior to *T. cruzi* infection may increase parasitemia and cardiac parasitism, also influencing NO production and cytokines (Lovo-Martins et al., 2018). In the same line, Torrecilhas et al. showed that mice pretreated with EVs before infection exhibited accelerated and increased mortality rates and developed severe heart pathology (Trocoli Torrecilhas et al., 2009).

On the other hand, using a different approach, it has been reported that EV inoculation increases the proportion of small peritoneal macrophages (SPM) over large peritoneal macrophages (LPM). Although this represents an alternative classification of macrophages, distinct from the conventional M1/M2 polarization, the induction of both pro-inflammatory and regulatory mediators supports the notion that EVs modulate the immune response (Cornet-Gomez et al., 2023a). Moreover, sialylated and non-sialylated IgG antibodies forming immune complexes with EVs differentially modulate macrophage activation through FcγR engagement. In this regard, sialylation of IgG alters the antibody structure in a way that shifts recognition from the pro-inflammatory FcRγ to the anti-inflammatory FcRγIIb. Consistent with this, SPMs and LPMs stimulated with sialylated IgG immune complexes produce cytokines associated with an anti-inflammatory profile, whereas stimulation with non-sialylated IgG immune complexes results in pro-inflammatory cytokine production (Cornet-Gomez et al., 2023a).

In addition to the effect of the parasite EVs on immune mechanisms or cells, the infection itself modulates host cell-cell communication (Ansa-Addo et al., 2024). Altogether, these findings uncover a novel layer of complexity in the regulation of the immune response by *T. cruzi* EVs or cell-infected EVs, with potential implications for both the acute and chronic phases of infection (Cornet-Gomez et al., 2025).

Another mechanism employed by *T. cruzi* to influence polarization is the induction of apoptosis in several cell types (Decote-Ricardo et al., 2017). Apoptosis of T cells, B cells, and neutrophils has been described (Cardoso et al., 2015; Cabral-Piccin et al., 2016; Decote-Ricardo et al., 2017; Magalhães et al., 2017). Uptake of apoptotic cells is a specialized and highly conserved form of phagocytosis termed efferocytosis (Freire-de-Lima et al., 2006). The involvement of the Axl macrophage receptor during efferocytosis induced by *T. cruzi* has been reported (Rigoni et al., 2022). This process leads to the production of anti-inflammatory cytokines such as IL-10, PGE_2_, and TGF-β by phagocytes (Fadok et al., 1998; Freire-de-Lima et al., 2006; Elliott et al., 2017), all of which are mediators that would favor the Th2-type polarization of macrophages. Moreover, it was reported that Injection of apoptotic bodies markedly increases parasitemia via PGE2 (Freire-de-Lima et al., 2000) and TGF-β, both of which suppress proinflammatory eicosanoid and NO synthesis (Freire-de-Lima et al., 2006).

One notable strategy used by the parasite consists of the expression of phosphatidylserine (PS) on its surface (Damatta et al., 2007). The interaction of parasite-derived PS with PS receptors on macrophages has been shown to subvert an important point of control, as it elicits a response associated with the phagocytosis of apoptotic cells, which inhibits the production of NO and the inflammatory response required to eliminate the parasite (Coria-Paredes et al., 2025).

LBs from macrophages are sites for PGE_2_ synthesis by COX-2, and as previously mentioned, PGE_2_ together with TGF-β may favor a Th2-polarization benefiting parasite replication (de Almeida et al., 2018).

Finally, thromboxane A2 (TBXA_2_) may also influence macrophage function. As much as 90% of circulating TBXA_2_ is thought to originate from the parasite and may influence phagocytes by inhibiting the effects of inflammatory cytokines such as TNF-α (Ashton et al., 2007).

## 3 Core 2. Avoiding the triggering of a Th1 response by dendritic cells

### 3.1 Introduction

The mononuclear phagocytic system, including monocytes, DCs, and tissue-specific macrophages, plays a central role in pathogen recognition and elimination. Plasticity is a signature of the myeloid compartment, and the particular on/off activation status of these cells makes them a perfect target for the pathogen evasion mechanisms. Basic information about DCs can be found in supplementary table III. Most *T. cruzi* studies have involved cDCs, and accumulating evidence shows that defined mechanisms can elicit different responses depending on the target cell type (Gravina et al., 2013; Gutierrez et al., 2020).

### 3.2 Infection and DC manipulation: from *in vitro* results to *in vivo* experience

Although less studied, it is likely that *T. cruzi* could infect DCs passively by phagocytosis or actively, using similar components as those described for macrophages and other cells (Scharfstein et al., 2000; Poncini et al., 2008; Caradonna and Burleigh, 2011; Morán-Utrera et al., 2012; Gil-Jaramillo et al., 2016; Lonien et al., 2017).

Evidence also shows that *T. cruzi* can disrupt normal DC function, delaying or dampening the immune response that should be mounted against an intracellular pathogen. In 1999, Van Overtvelt et al. reported that human monocyte-derived DCs can be infected by *T. cruzi* and that both infection and treatment with *T. cruzi*-conditioned medium (TCM) prevented an optimal maturation of DCs by LPS, since a reduced expression of HLA-DR and CD40 molecules was observed (Van Overtvelt et al., 1999). In addition, IL-12 and TNF-α secretion by DCs was significantly decreased after infection (Van Overtvelt et al., 1999).

In the same line, Alba Soto et al. (2003) and Planelles et al. (2003) described that costimulatory molecules are downregulated in splenic DCs after infection (Alba Soto et al., 2003; Planelles et al., 2003). Moreover, DCs from infected mice showed a decreased ability to stimulate CD4 and CD8 alloresponses (Alba Soto et al., 2003).

Interestingly, mouse strains with different susceptibility to the infection displayed differences in spleen DC activation. Susceptible mice displayed a lower rate of CD40 and CD86 expression, and decreased allostimulatory capacity compared to DCs from a more resistant strain (Planelles et al., 2003).

It has also been reported that intraperitoneal infection can limit DC migration/maturation, impairing the upregulation of CD86 (Chaussabel et al., 2003). In addition, *T. cruzi* infection caused a downregulation of the CD8α+ sDCs, a subtype specialized in cross-presentation (Van Overtvelt et al., 2002). *In vitro* studies demonstrated that bone marrow-derived DCs (BMDCs) cultivated with trypomastigotes acquired tolerogenic properties, preserving endocytic capacity, expressing low levels of costimulatory molecules (CD40, CD80 and CD86) and MHCII even in the presence of LPS. Most interestingly, tolerogenic DCs induced by the parasite secreted TGF-β, high levels of IL-10, and were poorly immunogenic (Poncini et al., 2008).

Comparison among *T. cruzi* strains suggests that while the extent of modulation varies, the overall effect is consistent. DC antigen-presenting and stimulatory functions are impaired, with increased IL-10 and PD-L1 expression and reduced IL-12 production (da Costa et al., 2014). Importantly, IL-10-deficient DCs pulsed with parasite antigens protect against lethal infection, enhancing Th1 response and antigen-specific T cell expansion (Alba Soto et al., 2010). Mechanistically, DC production of IL-10 is triggered through TLR4 signaling via ERK phosphorylation and NF-κB activation, and interestingly, this does not require infection but depends on parasite-DC contact (Poncini et al., 2010).

As previously mentioned, PRRs such as TLRs play a key role in eliciting effector immune responses, and some TLR knockout (KO) mice exhibit increased susceptibility to infection (Bento et al., 1996; De Arruda Hinds et al., 1999; Almeida and Gazzinelli, 2001; Oliveira et al., 2004, 2010; Medeiros et al., 2007; Caetano et al., 2011). However, GIPLs can target TLRs to interfere with macrophage and DC responses, benefiting the parasite (Brodskyn et al., 2002). The role played by GIPLs may be related to their structure, which is variable among strains (Carreira et al., 1996). For instance, ceramide-containing GIPLs are recognized by TLR4, while alkylacylglycerol GIPLs are recognized by TLR2/6 (Rodrigues et al., 2012). This variability probably confers different biological functions to this molecule, which may then affect macrophage and DC activation status, associated with the PRR repertoire displayed by each type of cell (Brodskyn et al., 2002; Barbosa et al., 2019).

Similarly, GPI anchor mucins that have shown pro-inflammatory effects (Ropert and Gazzinelli, 2000; Rodrigues et al., 2012) have also demonstrated an immunoregulatory role after interacting with TLR2 during *in vivo* infection (Campos et al., 2001; Rodrigues et al., 2012). This discrepancy may also be related to variability in host cell type —macrophages or DCs— and parasite strain (Gravina et al., 2013). In line with these findings, DCs with diverse origins —BMDCs in steady state and epidermis-derived DCs— responded differently to parasite infection and stimulation. Thus, available data provide evidence supporting the existence of a complex interplay between PAMPs, PRRs, and the proinflammatory or regulatory response elicited, which may depend on both parasite variability and the host cells’ type and status (Gutierrez et al., 2020).

Siglecs are sialic acid-binding Ig-like lectins that can inhibit immune cell activation in various species. Mouse Siglec-E is predominantly expressed on phagocytic cells (Zhang et al., 2004). *In vitro*, Erdmann et al. (2009) described that the Tulahuen strain exhibits higher TS activity and greater sialic acid coverage compared to the Tehuantepec strain (Erdmann et al., 2009). According to this, the authors showed that the Tulahuen strain can modulate DC function in a way that involves sialic acid and Siglec interaction. DCs incubated with Tulahuen parasites produced low IL-12 and higher levels of IL-10 compared to Tehuantepec. This effect was lost if parasites were desialylated or if the interaction was blocked by Siglec-blocking antibody (Erdmann et al., 2009). It is noteworthy that this pathway appears to be distinct from the TLR4-dependent IL-10 production by DCs described by Poncini et al. (2010), where small changes in the production of this cytokine were measured using desialylated parasites of the RA strain (Poncini et al., 2010).

The eicosanoid thromboxane A2 (TXA_2_) is another parasite mediator produced during infection (Nagajyothi et al., 2012). TXA_2_ was reported to notably influence DC function (Kabashima et al., 2003). Ashton et al. (2007) proposed that through its receptor (TP), TXA_2_ may negatively regulate dendritic cell–T cell interaction preventing maturation and antigen presentation (Ashton et al., 2007), and supporting the relevance of this interaction, TP null mice presented enhanced parasite load and mortality (Ashton et al., 2007).

Galectins (Gal) are endogenous lectins that can act as PRRs and modulators of innate and adaptive immune responses (Vasta, 2009). Both Gal-1 and Gal-3 were described to be upregulated during *T. cruzi* infection (Poncini et al., 2015, 2021; da Silva et al., 2017). A previous report showed that *T. cruzi* enhanced Gal-3 and Gal-3-specific ligands in splenic DCs (sDCs), suggesting implications in cell adhesion and migration (Chaussabel et al., 2003). Interestingly, *T. cruzi* Y strain can cleave the Gal-3 N-terminal domain, abrogating a microbicidal mechanism in the host (Pineda et al., 2020). Since N-terminal domain oligomerization is involved in some biological activities of this lectin, more studies are needed to define the scope of this mechanism in DCs. In the same line, under certain conditions, Gal-1 can act as a negative immune regulator. Gal-1-exposed DCs acquire tolerogenic properties and drive immunoregulatory T cell responses (Ilarregui et al., 2009). *T. cruzi* was described to trigger Gal-1 expression in BMDCs *in vitro* and during infection. In addition, it was also demonstrated the relevance of this mechanism in the tolerogenic profile elicited by trypomastigote-stimulated-DCs, since *Lgals1^-/-^* DCs remained immunogenic under parasite interaction (Poncini et al., 2015).

Another mechanism described for *T. cruzi* with impact on DC biology was reported for NKs in the regulation of the innate immune response by secreting IL-10 and controlling the survival of immature DCs. The extent of this mechanism would vary according to the virulence of the *T. cruzi* strain (Batalla et al., 2013). More recently, it was described that the parasite induced a heterogeneous myeloid cell infiltrate at the site of infection, including cells that resemble monocyte (Mo)-derived in the experimental acute infection. These Mo-DCs like cells, appear to display a dual role during acute infection by controlling parasitemia fostering inflammatory mechanisms, but impairing strong antigen presentation (Poncini and González-Cappa, 2017). A recent report re-evaluated the role of Mo-DCs in mice and proposed the existence of a population of inflammatory cDC2 sharing phenotypic features with Mo-DCs (Coillard and Segura, 2021). In consistency with the results described by Poncini & González Cappa (2017) they all showed the appearance of DCs compatible with inflammatory cDC2 (CD11b^+^Ly6C^+^CD64^+^CCR2^−^) that express the transcription factor Zbtb46 during acute infection (Poncini and González-Cappa, 2017). Nevertheless, real differences among Mo-DCs and inf cDC2 in the context of *T. cruzi* infection need to be addressed. In addition, a novel study using RNA-sequencing-based transcriptome analysis in human Mo-DCs in contact with metacyclic trypomastigotes *in vitro* showed the activation of pathways typically associated with viral infections (Gil-Jaramillo et al., 2021), further opening new perspectives for the study of the complex proinflammatory or tolerogenic network triggered by the parasite in cells from the phagocytic mononuclear system.

## 4 Core 3. Resisting and subverting the host complement system

### 4.1 Introduction

To establish a successful infection, pathogens entering the bloodstream must overcome the complement system, an essential part of the innate immune response that is composed of over 40 plasma and surface proteins. Basic information about the complement system can be found in supplementary table III. In general terms, complement activation ends with the effective elimination of invading microbes. Nonetheless, a select group of pathogens, including *T. cruzi*, have evolved sophisticated mechanisms to resist, evade, or even subvert this system to support their survival and persistence within the host (Ermert et al., 2019).

### 4.2 Complement resistance

*T. cruzi* possesses an extensive arsenal of proteins that directly counteract complement activation. Notably, the CPath is not efficiently activated by *T. cruzi* PAMPs (Cestari and Ramirez, 2010). As a result, during the first encounter between the parasite and host, the order of complement pathway against *T. cruzi* should be: LPath, APath, and finally CPath, as previously described (Cestari et al., 2013). By delaying CPath activation, the parasite gains valuable time to establish infection before humoral immunity is engaged.

The mechanisms used by *T. cruzi* to resist the LPath mainly consist of preventing the assembly of the C3 convertase, using proteins such as TcCRIT, a parasite receptor that binds to C2 and inhibits its cleavage by MASP (preventing C3 convertase formation), and T-DAF (Trypanosoma-decay accelerating Factor), which binds to C3b and C4b and likely accelerates the dissociation of the C3 convertase (Cestari et al., 2013). In addition, *T. cruzi* possesses a protein called CRP (complement regulatory protein) that belongs to the trans-sialidase family and that has a similar ability to T-DAF to dissociate the C3 convertase. The importance of CRP was shown by studies describing that epimastigotes engineered to overexpress this protein are more resistant to complement-mediated lysis (Norris, 1998).

On the other hand, it has been reported that *T. cruzi* can avoid the activation of the APath using a gp58/68 protein that inhibits the assembly of the alternative C3 convertase, likely by impairing the function of FB (Lidani et al., 2017). Additionally, the active trans-sialidase allows the parasite to cover its surface with sialic acid, also inhibiting to some extent the activation of the Apath (Ramírez-Toloza and Ferreira, 2017).

These mechanisms are reinforced through the generation of both parasite and host EVs. *T. cruzi* surface molecules such as oligopeptidase B and gp82 may trigger a transient increase in intracellular Ca^2+^ in host cells, leading to the release of EVs (Ramírez-Toloza and Ferreira, 2017). Since EVs carry host complement receptors like CR1 and DAF, they can contribute to inhibiting C3 convertase activity (Cestari et al., 2012).

These events may depend on the strain, since, for example, two *T. cruzi* strains from the TcI lineage, one with lower virulence (Ninoa) and another more virulent (Qro), differ in their resistance to the lytic activity of the complement system, which results in a reduced ability of the Ninoa strain to invade mammalian cells. This correlates with marked differences in the expression of these proteins (Arroyo-Olarte et al., 2018).

### 4.3 Subversion of the host complement pathway

*T. cruzi* not only employs strategies to resist and escape the complement system but also has evolved the capacity to take advantage of the mechanisms involved. For instance, EVs may facilitate host cell invasion via the lysosome-independent route in a way that relies on TGF-β exposed in the host EVs (Cestari et al., 2012).

Additionally, the parasite can capture fluid-phase host complement regulatory protein (FH), a negative APath regulator, and exploit its negative control of the APath to evade complement activation (Ramírez-Toloza and Ferreira, 2017).

Another mechanism exploitation of the host complement system by *T. cruzi* involves the TcCRT protein, which binds to C1q, promoting the uptake of the parasite by the host’s phagocytic cells, mimicking the clearance of apoptotic cells (Ramírez et al., 2011).

### 4.4 Buying time for infection

Although the CPath could only participate lately to cope with *T. cruzi* infection, it has been shown that this pathway would be important if it were activated early. Indeed, anti-galactosyl antibodies from Chagas disease patients have been shown to mediate complement-mediated lysis of *T. cruzi* trypomastigotes (Almeida et al., 1991).

During the acute phase, the parasite would delay the elicitation of the CPath activation targeting the cells required to produce the specific antibodies. In this sense, there is strong evidence that *T. cruzi* notably prevents the generation of a protective B-cell response by mechanisms that include unspecific B-cell activation, B-cell anergy and apoptosis (Bermejo et al., 2011; Toro Acevedo et al., 2017). It was reported that even up to 98% of activated B cells produce unspecific antibodies during the acute phase of *T. cruzi* infection (Minoprio, 2001). The main unspecific mitogens described to date are a proline racemase (PR) (Reina-San-Martín et al., 2000), the C-terminal repetitive motif of trans-sialidase proteins, called shed acute phase antigen (SAPA) (Gao et al., 2002), and Tc24 superantigen (Gunter et al., 2016).

A PR of 45 kDa is only expressed and released by infective metacyclic and bloodstream forms of the parasite. It has been implicated in non-specific polyclonal activation of B cells, an event that would prevent the development of effective immune responses against the parasite (Chamond et al., 2005).

Regarding SAPA, it was described that the C-terminal fragment of trans-sialidase is capable of activating B cells in a T-cell independent manner that leads to polyclonal antibody secretion (Gao et al., 2002).

On the other hand, Tc24 was shown to elicit a nonspecific B cell response secreting mainly IgM both *in vivo* and *in vitro*. Moreover, supporting a superantigen role of Tc24, it was shown that the injection of the antigen into athymic mice induced B-cell activation independent of T cell help (Gunter et al., 2016).

A report has described the existence of a repetitive fragment of the ribosomal protein L7a,(TcRpL7) (Toro Acevedo et al., 2017) that may function in suppressing B cell proliferation, which would be another mechanism impairing the elicitation of a protective B cell response.

Beyond functional distraction, *T. cruzi* also induces B cell death. Apoptosis has been observed both in the bone marrow and peripheral compartments (Acosta Rodriguez et al., 2007). In this sense, stimulation with a cyclooxygenase product released by *T. cruzi*-infected CD11b+ cells caused apoptosis of immature B cells in the bone marrow (Zuniga et al., 2005). Parasite-derived TXA2 has been proposed as a key mediator of this process (Ashton et al., 2007).

## 5 Core 4. Subversion of MDSCs

### 5.1 Introduction to role of neutrophils and monocytes during *T. cruzi* infection

Basic information about neutrophils and monocytes can be found in supplementary table III. It was reported that in models of natural transmission through skin, a low/moderate inflammatory reaction may occur, and neutrophils are recruited to the entry site (Monteon, 2019). On the other hand, in a model of skin infection, Poncini and González-Cappa (2017) reported that *T. cruzi* inoculation in the ear skin induced a low and focal mobilization of total leukocytes (Poncini and González-Cappa, 2017). An increased number of Gr-1^+^Ly6C^+^ dermal cells, compatible with monocytes, was observed at 24 hours post-infection. Strikingly, despite neutrophils constituting a high proportion of leukocytes in both mice and humans, it has been described that these cells decrease significantly in the blood and are absent from the infection site as early as 96 hours post-infection (Melo, 2009; do Carmo et al., 2017; Monteon, 2019). Supporting this observation, it was reported that *T. cruzi* interaction with neutrophils may cause apoptosis and NET (neutrophil extracellular trap) formation, a process that may account for the decrease of these cells early during infection (Sousa-Rocha et al., 2015; Magalhães et al., 2017).

On the other hand, it has to be considered that neutrophils may play different roles depending on the mouse strain infected. BALB/c mice depleted of neutrophils before infection showed exacerbated disease associated with lower components of a Th1 response, whereas neutrophil depletion in C57BL/6 mice increased resistance to infection in a manner compatible with the generation of an enhanced Th1 response (Chen et al., 2001). Overall, the evidence suggests that neutrophils rapidly reduce during the acute phase of infection, and their precise role in parasite control versus disease promotion remains incompletely understood.

Regarding monocytes, during *T. cruzi* infection, these cells have been shown to migrate to the heart and extravasate, where they can differentiate into macrophages and dendritic cells (Melo, 2009; Poncini and González-Cappa, 2017).

*In vitro*, parasite-derive tGPI-mucins induced IL-12 production by human blood monocytes, in a CD40-CD40L and IFN-γ-depended manner (Abel et al., 2014). However, despite this potential beneficial effect, it has also been reported that *T. cruzi* can cause the release of EVs from monocytes in a way that promotes parasite infection (Cestari et al., 2012).

It should be considered that genetic variability and different hosts may strongly affect the immune response elicited. Some strains of *T. cruzi*, such as Col cl1.7, may cause an *in vitro* increase of costimulatory molecules in monocytes, while other strains, such as Y strain, may not affect significantly those cells (Magalhães et al., 2019).

### 5.2 MDSCs vs neutrophils and monocytes

During pathological conditions involving excessive inflammation, the normal pool of peripheral myeloid cells may be severely affected, leading to rapid expansion of immature myeloid cells in the bone marrow that are subsequently exported to the periphery in a process known as emergency myelopoiesis. The current consensus is that mobilized immature myeloid cells are blocked in their differentiation and become suppressive when exposed to inflammatory mediators, termed myeloid-derived suppressor cells (MDSCs) (Gabrilovich and Nagaraj, 2009; Millrud et al., 2017). Currently, the nature and biological role of MDSCs have become clearer, and MDSCs have emerged as universal regulators of immune function during cancer, infections, and inflammation (Bronte et al., 2016; Dorhoi and Du Plessis, 2017; Medina and Hartl, 2018; Cabrera and Marcipar, 2019; Dorhoi et al., 2019; Veglia et al., 2021). MDSCs consist of at least two major groups of cells: granulocytic or polymorphonuclear MDSCs (PMN-MDSCs) and monocytic MDSCs (M-MDSCs). PMN-MDSCs are phenotypically and morphologically similar to neutrophils, whereas M-MDSCs are similar to monocytes (Gabrilovich and Nagaraj, 2009; Veglia et al., 2021). Despite phenotypic similarities, MDSCs represent a relatively stable, distinct state of functional activity of neutrophils and monocytes (Gabrilovich, 2017).

Neutrophils and PMN-MDSCs share the same origin and many morphological and phenotypic features. In mice, these cells have a phenotype of CD11b+ Ly6G+ Ly6C^low^. In humans, they are defined as CD11b+ CD14− CD15+/CD66b+ cells. Although the distinction between PMN-MDSCs and neutrophils is still controversial, transcriptome analysis supports that these populations represent two distinct myeloid subsets with different molecular, biochemical, and functional characteristics, with the suppressive capacity of PMN-MDSCs being the most notable difference (Fridlender et al., 2012; Veglia et al., 2018; Zhou et al., 2018). Finally, a subset of suppressive, IL-10-producing neutrophils has been reported in the context of *T. cruzi* infection (Tosello Boari et al., 2012).

In contrast to neutrophils, that have been described to decrease rapidly during acute *T. cruzi* infection (Melo, 2009; do Carmo et al., 2017; Monteon, 2019), it has been reported that blood monocytes increase during this stage (Melo, 2009; do Carmo et al., 2017). These increases could be committed to populating infected tissues with more macrophages and DCs. However, it is also possible that these cells, with the same phenotype as monocytes, also comprise cells belonging to the M-MDSC population, as increases of this subset of MDSCs have been reported during the acute phase of infection (Cuervo et al., 2011; Arocena et al., 2014; Millrud et al., 2017; Prochetto et al., 2017; Gamba et al., 2021). Alternatively, it was suggested that M-MDSCs may differentiate from monocytes by a reprogramming under inflammatory conditions (Millrud et al., 2017).

Although better markers are needed to distinguish monocytes and M-MDSCs, there exist differences in phenotype and function between these populations (Veglia et al., 2018). In mice, both monocytes and M-MDSCs express the following markers: CD11b+ Ly6G- Ly6C+, while in humans the HLA-DR is useful to distinguish both populations: monocytes express CD11b+ CD14+ CD15- HLA-DR+ but M-MDSCs express CD11b+ CD14+ CD15- HLA-DR^low/-^ (Veglia et al., 2018).

In general terms, monocytes mobilized under emergency myelopoiesis may have an immature phenotype and morphology that is compatible with M-MDSCs; displaying relatively weak phagocytic activity; increased background levels of reactive oxygen species (ROS), nitric oxide (NO) production, high expression of arginase, PGE_2_, and several anti-inflammatory cytokines (Veglia et al., 2018). In coincidence with PMN-MDSCs, immunosuppressive capacity remains the most reliable feature to distinguish M-MDSCs from monocytes. Both MDSC subsets deploy overlapping suppressive mechanisms, including not only ROS, NO, and PGE2, but also arginase, TGF-β, IL-10, carbon monoxide (CO), indoleamine 2,3-dioxygenase (IDO), heme oxygenase-1 (HO-1), and depletion of cysteine, thereby dampening T cell activation and effector (Gabrilovich, 2017; Millrud et al., 2017; Veglia et al., 2018).

### 5.3 MDSC manipulation

The first report linking *T. cruzi* infection to MDCS expansion was published in 2002, when Goñi et al. demonstrated that acute infection caused a notable increase in CD11b+Ly6G+(Gr-1+) cells, reaching up to 20% of the total spleenocytes. These cells expressed iNOS, produced NO, and suppressed T cell proliferation *in vitro*. Interestingly, MDSC expansion was abolished in IFN-γ KO mice, indicating that the induction of MDSCs depends on inflammatory signals (Goñi et al., 2002; Gabrilovich, 2017). Since then, several studies have confirmed that *T. cruzi* infection drives the accumulation of both PMN-MDSCs and M-MDSCs in multiple organs, including the spleen, liver, and the heart (Cuervo et al., 2011; Arocena et al., 2014). Their suppressive, mechanisms include the production of ROS, arginase-1, iNOS, and peroxynitrites (Cuervo et al., 2011; Arocena et al., 2014).

The most accepted model for development, expansion, and differentiation of MDSCs from a common myeloid progenitor is based on two signals (Gabrilovich, 2017). The following factors may contribute to the first signal that is necessary for development and expansion of MDSCs: GM-CSF, G-CSF, M-CSF, IL-6, adenosine, STAT3, IRF8, C/EBPβ, Notch, and others (Condamine et al., 2015). Then, inflammatory signals and several factors may be important for the acquisition of the suppressive capacity, such as IFN-γ, IL-6, PGE_2_, TNF-α, IL-1β, STAT1, STAT6, and others (Condamine et al., 2015). Strikingly, *T. cruzi* infection leads to the production of numerous factors that are required for MDSC expansion and activation, such as GM-CSF (Olivares Fontt et al., 1996), G-CSF (Silva et al., 2018), IL-6 (Cheng et al., 2011; Arocena et al., 2014), STAT3 (Arocena et al., 2014), IFN-γ (Torrico et al., 1991; Goñi et al., 2002; Bontempi et al., 2015; Gamba et al., 2021), PGE_2_ (Celentano et al., 1995; Abdalla et al., 2008; Mukherjee et al., 2011; de Almeida et al., 2018), IL-1β (Roggero et al., 2002), TNF-α (Roggero et al., 2002), and IL-17 (Tosello Boari et al., 2012; Cai et al., 2016), generating a milieu that is highly compatible with the notable increase of MDSCs observed during the acute phase of infection (**Figure 1**).

Notably, since PMN-MDSCs and M-MDSCs are barely detected in a basal state, it could be assumed that only mature neutrophils and monocytes would be involved in the processes that take place during the very early steps of *T. cruzi* infection. However, as the inflammatory state develops, and the factors required for MDSC expansion accumulate, the possibility exists that emergency myelopoiesis tends to replace neutrophils with PMN-MDSCs and monocytes with M-MDSCs in order to avoid an inflammatory condition incompatible with host survival (Goñi et al., 2002; Arocena et al., 2014; Prochetto et al., 2017; Gamba et al., 2021; Borgna et al., 2024). In support of this scenario, it was shown that MDSC depletion severely decreased mice survival, likely due to the exacerbation of the inflammatory response, as has been suggested (Arocena et al., 2014).

The notable increase of MDSCs in several organs may significantly affect other immune populations, exerting a substantial influence on the outcome of the infection. Although MDSC depletion during the acute phase of infection severely increased mortality to almost 100% (Arocena et al., 2014), studies using vaccinated mice treated with 5-Fluorouracil (5FU) have provided insights into their immunoregulatory role (Gamba et al., 2021). Interestingly, vaccinated mice that were depleted of MDSCs showed a stronger CD8 response in the spleen compared to vaccinated but non-MDSC-depleted mice, suggesting that even in the context of a vaccine with protective capacity, the remaining MDSCs still attenuate the expected effector response (Gamba et al., 2021). In addition, MDSC depletion correlated with an increased number of Foxp3^+^ Treg cells in the spleen, suggesting the existence of a balance between these populations of the regulatory arm of the immune system (Fresno and Gironès, 2018, 2021; Cabrera and Marcipar, 2019). Interestingly, CD8 responses were strengthened despite the rise in Tregs, highlighting that MDSCs may exert dominant suppression of effector T cells in acute infection. Additionally, the possibility exists that MDSCs increase to a level high enough to also suppress Tregs, a phenomenon that has been described in some scenarios (Centuori et al., 2012; Ji et al., 2016).

Recently, MDSCs have been shown to kill DCs in a NO-dependent manner (Ribechini et al., 2019). Although this mechanism has not been studied in the context of *T. cruzi* infection, MDSC depletion in vaccinated and infected mice enhanced DC activation markers, suggesting that MDSCs may also affect DCs during *T. cruzi* infection (Gamba et al., 2021; Borgna et al., 2024).

## 6 Core 5. Suppressing and delaying the adaptive immune response

### 6.1 Introduction

A central target of *T. cruzi* immune manipulation is to prevent the elicitation of the effector response. For this purpose, the parasite employs several strategies that affect the efficient function of both the innate and adaptive responses. Regardless of the mechanism employed, an important proportion of the affected pathways generates molecules that converge to prevent the correct activation of T and B cells. There is evidence that at least the following cells and components may play a role in impairing an efficient adaptive immune response:

### 6.2 Host components

#### 6.2.1 Arginase-1

L-arginine is a semi-essential amino acid crucial for T-cell activation and memory T cells (Rodriguez et al., 2003; Geiger et al., 2016a). In the context of *T.cruzi* infections, MDSCs and macrophages can express arginase-1 in response to TGF-β, IL-4, and IL-13, thereby depleting local L-arginine levels (Peluffo et al., 2004; Cerbán et al., 2020). Additionally, in the presence of inflammatory cytokines such as IFN-γ and TNF-α, arginase may be diverted toward NO production via inducible iNOS, further reducing its availability for T-cell (Giordanengo et al., 2002; Cuervo et al., 2008).

This L-arginine depletion has been linked to suppressed T-cell activation in infected hearts, allowing parasite multiplication (Rodriguez et al., 2003; Cuervo et al., 2008, 2011; Geiger et al., 2016b).

Strikingly, supplementation of L-arginine during infection has been shown to reduce parasite burden in heart tissue, confirming the functional importance of arginine deprivation in dampening adaptive immunity (Cuervo et al., 2011; Carbajosa et al., 2018).

#### 6.2.2 ROS

ROS, including superoxide (O_2_•^−^), hydrogen peroxide (H_2_O_2_), and hydroxyl radical (OH•), are small, short-lived, highly reactive molecules generated primarily by NADPH oxidase 2 (NOX2) in activated phagocytes (Belikov et al., 2015).

NOX2 utilizes NAD(P)H as an electron donor to reduce O2 to O_2_•^−^, which is then dismutated into other oxidants (e.g., H_2_O_2_, OH•) (Dhiman and Garg, 2011). ROS can affect neighboring T cells by modulating key signaling molecules, ultimately impairing proliferation and effector functions (Cemerski et al., 2002; Belikov et al., 2015).

In the context of *T. cruzi* infection, although the influence of ROS is usually associated with the oxidative burst of phagocytes to kill the parasite, ROS from macrophages or MDSCs may also participate by influencing the elicitation of the effector response. Supporting this role, Arocena et al. reported that the addition of a ROS scavenger to a culture of *T. cruzi*-infected splenocytes was able to partially restore the proliferative response (Arocena et al., 2014).

Finally, care must be taken concerning the role of ROS in adaptive immunity, as it has also been reported that NOX2 activity in macrophages is necessary for the development of a protective CD8 response (Dhiman and Garg, 2011).

#### 6.2.3 NO

At low concentrations (nanomolar range), NO acts as an intercellular messenger in several functions, including neurotransmission, vasodilation, inhibition of platelet aggregation, and modulation of leukocyte adhesion (Singh et al., 2000). Nonetheless, under inflammatory conditions and in the presence of inflammatory cytokines such as TNF-α, IFN-γ, and IL-12, high levels of NO (micromolar range) may be generated following iNOS induction in cells like macrophages, neutrophils, and MDSCs (Gutierrez et al., 2009; Veglia et al., 2018).

Similarly to ROS, NO is generally studied in the context of the oxidative burst against pathogens, but high levels of NO can induce thymocyte as well as splenic T-cell apoptosis (Okuda et al., 1996), and may affect the immune profile of Th cells, being unfavorable to the elicitation of the Th1 subset (Singh et al., 2000). Moreover, in the context of oxidative stress, NO can react with superoxide to form peroxynitrite, which can inhibit the proliferation and effector function of T cells (Gabrilovich et al., 2012; Kim et al., 2018). Interestingly, up to 70% of infected splenic MDSCs may produce peroxynitrite, resulting in extensive nitration of surface proteins on CD4^+^ and CD8^+^ T cells (Arocena et al., 2014), thereby directly impairing their activity.

#### 6.2.4 TGF-β

Although three isoforms of TGF-β with similar functions have been identified in mammals — TGF-β1, -β2, and -β3 — in fact, TGF-β1 is the main isoform expressed by cells of the immune system (called herein TGF-β). TGF-β is synthesized in an inactive form as a precursor linked to latency-associated protein (LAP) or latent TGF-β-binding protein (LTBP), which keeps TGF-β inactive (Travis and Sheppard, 2014). In the presence of an appropriate stimulus, the active form of TGF-β is produced after proteolytic processing. Then, the dissociated TGF-β can function as a cell surface-bound molecule or in a soluble form.

In general terms, TGF-β plays a very important role in processes related to T-cell development, tolerance, homeostasis, and differentiation (Li and Flavell, 2008). Concerning the influence of TGF-β on T cells, it is well known that this cytokine inhibits cytotoxic T lymphocytes and Th1 and Th2-cell differentiation, while promoting the generation of Treg, Th17, Th9, and Tfh cells (Waghabi et al., 2005; Li and Flavell, 2008; Travis and Sheppard, 2014).

At least macrophages, BMDCs, and Tregs may secrete TGF-β during *T. cruzi* infection (Poncini et al., 2008; Ambrosio et al., 2019; Cerbán et al., 2020). In addition, *T. cruzi* cruzipain may play a role in activating latent TGF-β (Waghabi et al., 2005; Ferrão et al., 2015), an event that then may influence not only cell invasion and macrophage polarization but also the adaptive immune response, impairing the elicitation of a Th1 profile and the activation of CD8+ T cells.

#### 6.2.5 IL-10

IL-10 is an anti-inflammatory cytokine that can inhibit the expression of MHC class II and the costimulatory molecules B7–1 and B7–2 on antigen-presenting cells, attenuating the elicitation of the effector response (Couper et al., 2008). In addition, it is well known that IL-10 can directly affect CD4+ T cells by inhibiting their proliferation and the production of many cytokines necessary for T-cell activation, such as IL-2, IFN-γ, and TNF-α (Joss et al., 2000). Several cells are able to produce IL-10, including macrophages, CDs, B cells, Tregs, and CD8 T cells (Moore et al., 2001).

According to its function, in the context of *T. cruzi* infection, IL-10 can prevent the development of an efficient effector immune response, which may favor the spread of the infection (Flávia Nardy et al., 2015). Although IL-10 plays an immunosuppressive role, it has also been shown that IL-10^-/-^ KO mice are not able to survive a challenge with *T. cruzi*, despite the production of high levels of serum TNF-α, IL-12, and IFN-γ, likely because of the generation of an exacerbated immune response (Hunter et al., 1997). In line with these results, the group of Alba Soto et al. also found that IL-10 KO-infected mice had higher parasitemia levels and mortality compared to WT-infected mice. Interestingly, the CD8^+^ effector response was reduced in IL-10 KO mice, as evidenced by a lower relative number of splenic and circulating CD8^+^ T cells and diminished effector functions (Pino-Martínez et al., 2019).

#### 6.2.6 SLAMF1-ligand

Signaling lymphocytic activation molecule family member 1 (SLAMF1/CD150) is a co-stimulatory receptor expressed on T cells, B cells, macrophages, and dendritic cells, regulating cell activation, proliferation, and survival (Sintes and Engel, 2011). *T. cruzi* exploits SLAMF1 to modulate host immunity by binding the receptor on macrophages, altering NADPH oxidase assembly and ROS production, and promoting parasite persistence. SLAMF1 engagement may also indirectly dampen adaptive immunity by limiting optimal T-cell activation and cytokine production. This dual mechanism, enhancing intracellular survival while suppressing effector responses, highlights SLAMF1-ligand interactions as a central strategy of immune subversion during infection (Poveda et al., 2020).

Immune-endocrine response

As previously noted, the ability of *T. cruzi* to persist and cause pathology appears to depend on multiple factors, including parasite strain, infective load and route of infection, presence of virulence factors, the parasite’s capacity to evade or subvert protective immune responses, the strength and nature of host defense mechanisms, and the host’s genetic background. Another important way in which *T. cruzi* may impair host responses or influence disease progression is by acting—either directly or indirectly—on supraphysiological immune regulatory systems, such as the neural and endocrine networks (Morrot et al., 2016; González et al., 2020). Some basic information regarding the hypothalamus–pituitary–adrenal (HPA) axis is provided in supplementary table III.

Certain pathogens, including *T. cruzi*, can exploit immunoregulatory processes to their advantage. Host–*T. cruzi* interactions have been shown to influence the course of infection by modulating various immunoendocrine axes (González et al., 2020). Comparative studies in *T. cruzi*-infected susceptible and resistant male mice have shown that disease susceptibility is influenced by the extent and timing of HPA axis activation (Roggero et al., 2006). In this regard, BALB/c mice -which can survive infection with the Tulahuen strain- display higher basal corticosterone levels, followed by an early increase in the hormone, whereas C57BL/6 susceptible mice exhibit a marked delay in initiating the anti-inflammatory response (Roggero et al., 2006). These findings suggest that timely anti-inflammatory signaling is essential for the parasite’s successful persistence within the host. In addition, since glucocorticoids influence numerous physiological processes, some of their actions are expected to be beneficial for the host. For example, endogenous glucocorticoids have been shown to support humoral immunity, which can enhance resistance to intracellular pathogens such as *T. cruzi* (Elenkov, 2004). Consistently, resistant mice develop an earlier *T. cruzi*-specific IgG antibody-response compared to susceptible strain (Pérez et al., 2005). These results are in line with studies on MDSCs that have reported that Tulahuen-infected BALB/c mice present higher MDSC levels and greater survival rates compared with infected C57BL/6 mice, which are more likely to succumb to an uncontrolled inflammatory response (Arocena et al., 2014).

In experimental models of acute *T. cruzi* infection, inflammatory cytokines such as TNF-α, IL-6, and IL-1β activate the HPA axis, leading to corticosterone secretion. Among these, IL-1β is the most potent activator of the HPA axis (Matsuwaki et al., 2014). Accordingly, the earlier and higher circulating levels of IL-1β observed in BALB/c mice correlate with a more effective activation of this neuroendocrine pathway (Roggero et al., 2002, 2006).

Another mechanism involving endogenous glucocorticoids that may favors parasite persistence relates to their impact on the thymus—the primary lymphoid organ responsible for the development and maturation of T cells, which are later exported to the periphery to shape the T cell repertoire (Savino et al., 2016). Elevated corticosterone levels during acute *T. cruzi* infection induce marked thymic atrophy, which may partially persist into the chronic phase (Roggero et al., 2002). Moreover, intrathymic endocrine circuits mediated by corticosterone and prolactin are disrupted during infection, promoting the loss not only of immature thymocytes but also of regulatory T cells (Pérez et al., 2020). In addition, *T. cruzi* infection induces multiple alterations in the thymic microenvironment, impairing normal T cell development and likely driving the abnormal, premature export of immature double-negative and double-positive thymocytes with a pro-inflammatory activation profile to the periphery (Pérez et al., 2020).

Given that *T. cruzi* can induce thymic atrophy and promote the release of non-selected, potentially autoreactive T cells, the possibility of defective negative selection during infection was investigated. However, negative selection of T cells bearing TCRs against self-antigens appears to remain intact in the thymus of infected animals, suggesting that central tolerance to self is not compromised. Notably, *T. cruzi* can infect the thymus, allowing its antigens to be presented in the context of MHC molecules and thereby engaging tolerance mechanisms. The intrathymic presence of *T. cruzi*-derived antigens could favor the generation of parasite-specific regulatory T cells, potentially contributing to immune tolerance toward the pathogen. If such a process indeed occurs, it may induce central tolerance to parasite antigens, ultimately weakening the development of effective protective immunity (Morrot et al., 2016).

Other tissues that can be directly affected by *T. cruzi* infection include key components of the neuroendocrine system. In this regard, the parasite can invade the hypothalamus–pituitary unit and the adrenal glands, leading to various functional consequences (Corrêa-de-Santana et al., 2006). *T. cruzi* can destroy infected neuroendocrine cells, thereby reducing glandular activity. In addition, the presence of parasites or their antigens within these glands may promote the recruitment of inflammatory cells and the local production of cytokines, which can modulate hormonal output—either enhancing or suppressing it—at the site of infection (Corrêa-de-Santana et al., 2006; da Silva Oliveira Barbosa et al., 2019). Furthermore, local inflammatory reactions can induce structural alterations, such as increased extracellular matrix deposition, which may further contribute to neuroendocrine dysfunction (Corrêa-de-Santana et al., 2006, 2009). In addition, we observed that in susceptible mice, the delayed glucocorticoid secretion occurring in the late phase of acute infection is clearly uncoupled from HPA–derived signals. Instead, an inflamed adrenal microenvironment appears to drive this response, through the emergence of intracellular mediators—such as PGE_2_, cAMP, and EPAC2—that participate in hormone synthesis and act as key triggers for corticosterone production (da Silva Oliveira Barbosa et al., 2019).

Another strategy employed by *T. cruzi* to enhance its persistence and evade immune detection is its ability to hide within adipose tissue. This tissue can be regarded as an immunoendocrine organ, as it produces a wide array of mediators collectively known as adipocytokines—some of which, such as leptin, function both as cytokines and hormones. Notably, studies have shown that adipose tissue may serve as a reservoir where dormant parasites can persist in a latent state, evading host defense mechanisms and potentially acting as a site of reactivation (Combs et al., 2005; Manarin et al., 2013; Lewis et al., 2014; Sánchez-Valdéz et al., 2018). Both *in vivo* and *in vitro* studies have shown that *T. cruzi* disrupts adipocyte catabolic and anabolic metabolism, largely due to robust downregulation of PPAR-γ, resulting in marked suppression of lipolytic and lipogenic enzyme expression during acute infection (González et al., 2019). Some authors have suggested that neuroendocrine and metabolic alterations induced by persistent *T. cruzi* infection may elevate the risk of developing diabetes, metabolic syndrome, and cardiovascular disease (Tanowitz et al., 2011; Brima et al., 2015; González et al., 2018; Rodeles et al., 2021), however this topic deserves more studies.

### 6.3 Host cells

#### 6.3.1 γδ T cells

Proinflammatory, but also regulatory, functions have been associated with γδ T cells (Lawand et al., 2017). In this sense, Cardillo et al. (1993) were the first to describe that γδ T cells are involved in the suppression of immune responses during the acute phase of *T. cruzi* infection (Cardillo et al., 1993). Recent reports describe that γδ T cells can be defined based on distinct cytokine profiles. For instance, IFN-γ–producing (γδT1) and IL-17–producing (γδT17) cells may constitute distinct functional phenotypes of γδ T cells (Korn and Petermann, 2012). Interestingly, it has been reported that γδ T cells secreting IL-17 are able to recruit MDSCs, which can mediate the immunosuppressive role of γδ T cells, as has been suggested not only in the context of T. cruzi infection but also during hepatitis B virus infection (Kong et al., 2014).

#### 6.3.2 IL-17 producing B cells

In addition to γδ T cells, B cells can also contribute as a source of IL-17 during acute *T. cruzi* infection (Bermejo et al., 2013). *T. cruzi* trans-sialidase influence on B-cell CD45 is responsible for IL-17 secretion by these cells in a non-canonical manner (Bermejo et al., 2013). Supporting a role of IL-17 in the immunosuppressive network elicited by *T. cruzi*, another study suggested that IL-17 may play a role in recruiting suppressive IL-10-producing neutrophils (Tosello Boari et al., 2012), an observation that is in line with the induction of MDSCs by IL-17 reported by the group of Cardillo et al. (1993).

In another correlation, it has been described that IL-10-suppressing neutrophils recruited by IL-17 are able to modulate IFN-γ production (Tosello Boari et al., 2012), and that deletion of IL-17-producing γδ T cells can result in a rise in IFN-γ levels (Cardillo et al., 2015). In both cases, the results suggest that IL-17 (produced by B cells or γδ T cells) may recruit MDSCs or IL-10-producing neutrophils.

#### 6.3.3 Treg Foxp3+ cells

The useful characterization of CD4+CD25+ Treg cells allowed the study of the role and mechanisms of suppression of this population in several pathological and non-pathological contexts (Belkaid et al., 2002; Sakaguchi et al., 2008, 2020). Currently, it is well known that in addition to its role in maintaining self-tolerance, Treg cells also play a role during pathogen infection and can be the target of several pathogens that subvert their regulatory function to persist in their hosts (Belkaid et al., 2002; Belkaid, 2007; Boer et al., 2015). We have previously reviewed the studies that addressed the influence of Tregs during *T. cruzi* infection (Cabrera and Marcipar, 2019). Although the studies differ in several parameters such as mouse strains used (BALB/c, C57BL/6, C3H/HeJ, A/J mice, outbred Swiss), DTUs of the parasite strains (Tulahuén, Y strain, Colombian, Brazil, Yucatán, RA), parasite doses (50, 100, 1,000, 2,000, 10,000, 80,000), inoculation routes (subcutaneous and intraperitoneal), and approaches to study Tregs (anti-CD25 depletion, adoptive transference, IL-2 + dexamethasone treatment, depletion of Tregs with diphtheria toxin), it could be hypothesized that Tregs may play a role in the suppressive network to favor host survival (when necessary), even if they participate in dampening the effector response, and favoring the persistence of the parasite (Sales et al., 2008; González et al., 2015; Araujo Furlan et al., 2018; Cabrera and Marcipar, 2019).

Different cells and molecules from the host and the pathogen have been described as influencing the Treg population during *T. cruzi* infection. TGF-β and IL-10 are among the most important host cytokines that may favor the Treg profile of CD4+ T cells.

### 6.4 Components of the parasite

#### 6.4.1 Tc52

Tc52 is a parasite protein that can be released by the parasite and that contains a tandemly repeated domain structure characteristic of glutathione S-transferases (Ouaissi et al., 1995). Since Tc52 can inhibit spleen cell proliferation *in vitro* and the addition of glutathione is able to restore the proliferative response, Tc52 may play an immunosuppressive role as a cysteine and glutathione scavenger both *in vitro* and *in vivo* during *T. cruzi* infection (Ouaissi et al., 1995; Fernandez-Gomez et al., 1998).

The relevance of the role of Tc52 has also been highlighted by Tc52-targeting experiments that showed that mutant parasites lacking even one Tc52 allele exhibited low virulence *in vivo* and *in vitro* (Allaoui et al., 1999).

On the other hand, it has also been shown that Tc52 may affect cytokine and NO production by macrophages, and cause DC maturation, increasing the expression of CD83 and CD86, as well as the production of IL-8, monocyte chemoattractant protein-1 (MCP-1), and macrophage inflammatory protein-1 (MIP-1) (Fernandez-Gomez et al., 1998; Ouaissi et al., 2002).

Taken together, Tc52 appears to play a dual role, activating immune effector functions while also restraining the response by depleting cysteine and glutathione.

#### 6.4.2 GIPLs

Among the multiple immunomodulatory effects of *T. cruzi* GIPLs, the ceramide fraction has been shown to be able to block *in vitro* CD4^+^ and CD8^+^ T-cell responses. This effect also includes inhibition of IL-2 secretion and reduced expression of CD25 on both CD4^+^ and CD8^+^ T cells (Gomes et al., 1996).

Interestingly, it has been reported that GIPLs are important B-cell-activating molecules, but without causing an increase in antibodies against themselves (DosReis et al., 2002).

#### 6.4.3 MASPs

MASP members constitute the second largest family of genes in *T. cruzi*. The large number of genes and the high degree of sequence variability among MASP members, together with their surface localization on infective forms of *T. cruzi*, are consistent with antigenic variation strategies and suggest that this multigene family participates in mechanisms of host–parasite interaction and immune evasion. For instance, it has been reported that *T. cruzi* metacyclic trypomastigotes previously incubated with IgG antibodies generated against a MASP protein of 52 kDa named MASP52 reduced the ability of parasites to invade Vero cells. Moreover, addition of the protein to inert particles led to endocytosis by non-phagocytic cells, providing strong evidence for a role in parasite invasion (De Pablos et al., 2011).

Additionally, due to the high quantity of MASP proteins, which are also secreted in EVs (De Pablos et al., 2016), it has been suggested that simultaneous expression of these proteins on the surface may act as a “smoke curtain”, confounding the immune system and preventing the mounting of an efficient immune response (Leão et al., 2022). More studies are required to better elucidate the role of MASP proteins in evasion of the immune system (Leão et al., 2022).

#### 6.4.4 B-cell nonspecific or nonprotective antibody inducers

It has been reported that a notable proportion of B cells — up to 98% — may produce unspecific antibodies during the early acute phase of *T. cruzi* infection (Minoprio et al., 1988; Minoprio, 2001). Some parasite proteins may be responsible for this effect, such as the proline racemase (PR) (Reina-San-Martín et al., 2000), the shed acute phase antigen (SAPA) (Gao et al., 2002) of the TS protein, and the Tc24 superantigen (Da Silva et al., 1998).

As a consequence of this nonprotective response, the development of an effective B cell response against the parasite is markedly delayed (Chamond et al., 2005).

#### 6.4.5 Suppressors of lymphocyte proliferation

AgC10 is a *T. cruzi* mucin found in insect and amastigote forms that has been shown to inhibit T-cell proliferation and block IL-2 transcription, an effect linked to surface CD62L binding and independent of NO and IFN-γ (Alcaide and Fresno, 2004). Additionally, the repetitive fragment of the ribosomal protein L7a (TcRpL7a) has been reported to suppress B-cell proliferation (Toro Acevedo et al., 2017).

#### 6.4.6 Trans-sialidase super family

Both active and inactive trans-sialidases are considered major virulence factors, either due to their capability to dampen host cell immunity or their ability to mediate the interaction between the parasite and the host (Ferrero-García et al., 1993; Freire-de-Lima et al., 2015; Campetella et al., 2020). Active trans-sialidases (aTS) belong to group I of the trans-sialidase/trans-sialidase-like family (TS). It is an enzyme that is exposed on the surface of the parasite but can also be released into circulation, causing additional effects (Freire-de-Lima et al., 2015). Sialic acid residues are present in all mammalian cells and exert crucial roles regulating both innate and adaptive immunity. Since aTS is able to transfer sialic acid between host cells—in addition to transferring sialic acid from the host to the parasite—several immune functions could be subverted by aTS and have been reviewed elsewhere (Flávia Nardy et al., 2015; Morrot et al., 2016). For instance, administration of TS modulated B and T lymphocytes (Freire-de-Lima et al., 2015), and even caused the apoptosis of thymocytes and mature T cells (Mucci et al., 2002, 2006). Moreover, aTS is able to sialylate the surface of CD8 T lymphocytes. This sialylation impairs the capacity of CD8 cells to interact with their cognate peptide in the context of MHC I. As a consequence, MHC I-dependent killing of *T. cruzi*-infected cells is decreased by aTS activity (Nardy et al., 2016). In addition, it has been postulated that SAPA and other TS epitopes may act by distracting and delaying the production of antibodies against the TS active site (Pitcovsky et al., 2002).

Trans-sialidases from the group II have been mainly associated with the invasion process, while genes from the group III are linked to the evasion of complement, as previously described (Freitas et al., 2011).

In conjunction, the large quantity of proteins belonging to this family supports the concept that such polymorphic proteins also function as a smoke screen, driving the generation of several non-protective antibodies (Cardoso et al., 2015).

## 7 Concluding remarks

More than a century after its initial description, *T. cruzi* remains a complex pathogen that challenges all efforts to neutralize it through immunological approaches. To date, no vaccine, prophylactic or therapeutic, has successfully advanced to late-phase clinical trials. Since it is a natural condition of the immune system to always act as a double-edged sword, eliminating autoreactive immune cells, dangerous microorganisms, and cancer cells while limiting damage to self, parasite infection inserts itself into the midst of a myriad of complex interactions among immune cells, which have evolved to cope with a wide range of dangers but can also be subverted by evolved pathogens that leverage the control mechanisms for their own benefit.

Thus, to further advance new approaches, it would be productive to integrate most of the knowledge acquired from years of research. The purpose of this review is to initiate a first attempt to integrate most of the known pathways exploited by *T. cruzi* to manipulate the host immune system. From the figure, it can be appreciated the extensive effort and the diversity of the strategies evolved by the parasite to prevent proper elicitation of the immune response. Four cores of manipulation are centered in the innate immune system, including incorrect macrophage polarization, impairment of cDC type I activation, complement interference, and MDSC subversion, all potent measures designed to avoid the mounting of an efficient adaptive immune response. In this sense, a proper B- and T-lymphocyte response is notably delayed, not only by interference with the innate immune response but also by providing a set of distracting antigens that shape the response against non-protective targets.

To date, numerous antigens have been evaluated as vaccine candidates and have been reviewed elsewhere (Freire-de-Lima et al., 2015; Rodríguez-Morales et al., 2015; Bivona et al., 2020; Dumonteil and Herrera, 2021; Teixeira et al., 2025). Interestingly, most of the antigens that have been extensively assessed, such as cruzipain, trans-sialidase, and non-specific B-cell mitogens, marked in bold in **Figure 1** and described in Supplementary Table II, participate in pathways involved in the manipulation of the host immune system.

One concept derived from the figure suggests that developing a vaccine based on a single antigen could be feasible to improve the infection outcome, but very challenging to achieve sterilizing protection, as the parasite employs overlapping strategies and does not rely on unique proteins to subvert the core mechanisms described in the text.

Conversely, it can be speculated that vaccines could be improved by increasing the number of antigens to prevent all core manipulations, but this alternative also faces different challenges. Since the immune response has limited resources and responds unequally to every epitope, a broad multiepitope vaccine may not necessarily improve vaccines and, after infection, the elicited response may inadvertently synergize with the nonspecific polyclonal effector response triggered by the parasite, potentially converging to elicit strong immunosuppression that suppresses responses directed toward protective antigens.

The fact that some parasite attenuated vaccines have reached sterilizing protection provides evidence that in some cases the use of a large quantity of antigens, covering all core manipulation, can eventually sum up to provide protection, but correlates of protection have not been determined in those studies.

Therefore, for safer vaccines not based on attenuated parasites, this review aligns with a framework of rational design that carefully optimizes combinations of protective antigens to prime effector responses. Additionally, controlling the generation of early nonspecific immune suppression could be another important goal to be achieved. In this manner, rationally optimizing immune targets may hold promise for advancing vaccine development, aimed at neutralizing the immune network manipulation by *T. cruzi*.
