# Supplementary material for: Towards a map of the immune system manipulation network by Trypanosoma cruzi
Source: Front Cell Infect Microbiol. 2026 Jan 6;15:1711520. doi: 10.3389/fcimb.2025.1711520 (PMC12816262; doi:10.3389/fcimb.2025.1711520)
Supplement: Supplementary file 1 [file Table1.docx]

**Supplementary Table I. Basic principles of life cycle and effector immune response against *T. cruzi***

|  | **Basic principles** |
| --- | --- |
| **Life cycle of *T. cruzi*** | During vectorial and oral transmission, the mammalian stage of the *T. cruzi* life cycle begins when metacyclic trypomastigotes from vector feces or contaminated food encounter skin lesions or reach the mucosal epithelium. These mTs infect both phagocytic and non-phagocytic cells near the site of entry. Regardless of the cell type infected, the parasites escape from the parasitophorous vacuole into the host cell cytoplasm, where they differentiate into amastigotes. After intracellular replication, amastigotes transform into bloodstream trypomastigotes. This form can induce host cell lysis to infect neighboring cells or enter the bloodstream to disseminate to distant tissues. Alternatively, bT can be ingested by a feeding vector, initiating the insect stage of the cycle. Once inside the digestive tract of the insect, trypomastigotes differentiate into epimastigotes, which multiply by binary fission. These epimastigotes then migrate to the rectum, where they differentiate into mT that are excreted with feces, thus completing the cycle by infecting a new vertebrate host (Acevedo et al., 2018; Lidani et al., 2019).  It has also been reported that an alternative sub-cycle may occur when amastigotes are released following host cell rupture, as they can be engulfed by macrophages, allowing their survival and subsequent differentiation into trypomastigotes (Ley et al., 1988). |
| **Effector immune response against *T. cruzi*** | Vertebrate infection by the vectorial or oral route begins when mT penetrates the host through lesions in the skin or through the oral mucosa. It is estimated that the initial inoculum can be small, ranging from hundreds to a few thousand parasites (Monteon, 2019). The first round of replication in situ would occur mainly in host tissues rather than in immune cells (Padilla et al., 2009; Cardoso et al., 2015). The parasite can invade virtually any nucleated cell in a process that may take 5–10 minutes after trypomastigotes attach to the host cell surface (Rodríguez et al., 1996).  Poor migration of the parasite to the draining lymph nodes, as well as limited mobilization of leukocytes to the skin, has been described (Cardoso et al., 2015; Poncini and González-Cappa, 2017).  *In vitro* observations suggest that *T. cruzi* may take 4-5 days to release the first round of progeny to nearby cells or bloodstream (Padilla et al., 2009). By consensus, this initial phase of infection has been described as relatively silent, without significant activation of the immune system (Padilla et al., 2009; Cardoso et al., 2015).  After the first round of replication, an encounter with the immune system becomes inevitable. Macrophages and dendritic cells (DCs) patrolling the tissues can recognize *T. cruzi* through several pattern recognition receptors (PRRs) that detect pathogen-associated molecular patterns (PAMPs). Total lipid extracts from the RA and K98 strains have been shown to induce a pro-inflammatory response via TLR2/6 in macrophages and HEK cells (Bott et al., 2018). Large quantities of glycoinositolphospholipids (GIPLs) are present covering the parasite as free molecules. Additionally, glycosylphosphatidylinositol (GPI) anchors attach glycoproteins and polysaccharides to the parasite surface (Almeida and Gazzinelli, 2001). It has been reported that GPI anchors derived from *T. cruzi* are ligands of TLR2; and induce the synthesis of pro-inflammatory cytokines such as IL-12, TNF-α and nitric oxide (NO) in innate immune cells (Almeida and Gazzinelli, 2001).  In addition, the lipid fraction of GIPLs, which shares a structure similar to LPS in some *T. cruzi* strains, is recognized by TLR4 and induces proinflammatory and immunomodulatory effects (Oliveira et al., 2004; Medeiros et al., 2007). Moreover, it has been described that *T. cruzi* GIPLs may activate NK cells and elicit antibody secretion from B cells (Bento et al., 1996; De Arruda Hinds et al., 1999).  On the other hand, it was reported that parasite RNA interacts with host TLR7 (Caetano et al., 2011), whereas unmethylated CpG DNA binds TLR9, a fact that may induce IL-12p40, TNF-α, and NO production by immune cells such as spleen DCs and bone marrow-derived DCs (Kayama and Takeda, 2010; Gravina et al., 2013; Acevedo et al., 2018). The relevance of some TLRs has also been shown using KO murine models for TLR4, TLR9, and MyD88 KO mice, which are more susceptible to *T. cruzi* infection (Campos et al., 2004; Bafica et al., 2006; Oliveira et al., 2010; Gravina et al., 2013). The pathways would not be individually essential, and the importance for the elicitation of the adaptive immune response should be better clarified (Oliveira et al., 2004). For instance, it has been shown that TLR2, TLR4, TLR9, and MyD88 KO mice are still able to elicit a CD8 effector response (Oliveira et al., 2010).  In accordance with the molecules secreted when the innate system interacts with *T. cruzi*, it has long been described that a protective immune response must include IFN-γ, IL-12, and NO. This observation is also supported by the fact that mice deficient in these immune components, associated with Th1 polarization, are highly susceptible to *T. cruzi* infection (Kayama and Takeda, 2010).  Macrophages cultured with *T. cruzi* are readily infected and lysed, whereas pretreatment of macrophages with IFN-γ decreases parasite infection by a mechanism that includes the expression of iNOS and the production of NO. In addition, TNF-α has been shown to play a synergistic role, potentiating the effect of IFN-γ (Muñoz-Fernández et al., 1992). In addition, after reacting with superoxide O_2_•−, NO may form the cytotoxic agent peroxynitrite, which could be crucial for killing *T. cruzi* (Alvarez et al., 2011; Koo et al., 2016).  The macrophage galactose-type C lectin (MGL1) receptor has been implicated in the recognition of *T. cruzi* and the activation of these cells, since macrophages from KO mgl1⁻/⁻ mice were heavily parasitized *in vitro* and showed decreased levels of reactive oxygen species (ROS), nitric oxide (NO), IL-12, and TNF-α (Rodriguez et al., 2020).  The relevance of IL-12 and IFN-γ in the induction of a Th1 cell response is reinforced by the role played by natural killer (NK) cells. It has been reported that NK cells are among the first cells to secrete IFN-γ and may even kill the parasite by a contact-dependent mechanism (Lieke et al., 2004; Sardinha et al., 2006).  A Th1 profile also accounts for the induction of a cytotoxic CD8 T cell response (Padilla et al., 2009). The importance of the CD8 response in controlling infection was also highlighted by the use of CD4 and CD8 T-cell knockout (KO) mouse models, which are highly susceptible to infection (Kumar and Tarleton, 1998). In addition, in recent years, a protective role for CD4 T cells with a Th17 profile has also been suggested (Cai et al., 2016).  Additionally, B cell responses were also reported to play a critical role (Kumar and Tarleton, 1998; Bermejo et al., 2011, 2013), although the appearance of protective antibodies (Ab) may not appear until the third week of infection (Minoprio et al., 1988; Bermejo et al., 2011). Anti-*T. cruzi* specific Ab would also be important for antibody-dependent, complement-mediated lysis, as it has been described that trypomastigotes are resistant to lysis in the absence of Ab (Lidani et al., 2017). Several surface targets, such as α-galactosyl (α-gal) epitopes, were suggested to allow antibody-dependent complement-mediated lysis (Gazzinelli et al., 1991; Krautz et al., 2000).  Interestingly, there are very few reports that highlight a role for DCs in activating the effector immune response. For instance, it was reported that the protein cruzipain of *T. cruzi* induces DC maturation through the activation of the bradykinin B2 receptor, an event that could be important for the elicitation of a protective Th1 response (Monteiro et al., 2007). In this sense, adoptive transfer of B_2_R^+/+^ DCs to B_2_R^-/-^ mice rescued Th1 and cytotoxic T cell responses, protecting the animals from the challenge of acute infection (Monteiro et al., 2007). Additionally, it was described that another parasite protein, Tc52, was able to induce maturation of DCs via TLR2 (Ouaissi et al., 2002). Furthermore, *T. cruzi* invasion leads to the activation of NFATc1 and DC maturation, resulting in IFN-γ production in a TLR-independent manner (Kayama and Takeda, 2010).  Collectively, several lines of research suggest that control of *T. cruzi* infection by the effector immune response depends on both innate and adaptive immunity, involving the complement system, NK cells, macrophages, DCs, T and B lymphocytes, and the production of proinflammatory Th1 cytokines such as IFN-γ, TNF-α, and IL-12, and eventually also IL-17 and the Th17 profile of CD4 T cells (Machado et al., 2012). Nonetheless, there is clear evidence that the effector response against *T. cruzi* cannot prevent the establishment of chronic infection (Minoprio et al., 1988; Cardillo et al., 1996; Padilla et al., 2009; Bermejo et al., 2011).  In addition, the fact that even vaccinated mice primed to better respond to infection are unable to generate sterilizing immunity, strongly supports that *T. cruzi* has evolved very sophisticated and successful strategies to manipulate the network of the host immune system for its benefit (Cardillo et al., 2015; Cardoso et al., 2015; Flávia Nardy et al., 2015; Morrot et al., 2016; Fresno and Gironès, 2018). |

Acevedo, G. R., Girard, M. C., and Gómez, K. A. (2018). The Unsolved Jigsaw Puzzle of the Immune Response in Chagas Disease. *Front. Immunol.* 9, 1929. doi: 10.3389/fimmu.2018.01929

Almeida, I. C., and Gazzinelli, R. T. (2001). Proinflammatory activity of glycosylphosphatidylinositol anchors derived from Trypanosoma cruzi: structural and functional analyses. *J. Leukoc. Biol.* 70, 467–477.

Alvarez, M. N., Peluffo, G., Piacenza, L., and Radi, R. (2011). Intraphagosomal peroxynitrite as a macrophage-derived cytotoxin against internalized Trypanosoma cruzi: consequences for oxidative killing and role of microbial peroxiredoxins in infectivity. *J. Biol. Chem.* 286, 6627–6640. doi: 10.1074/jbc.M110.167247

Bafica, A., Santiago, H. C., Goldszmid, R., Ropert, C., Gazzinelli, R. T., and Sher, A. (2006). Cutting edge: TLR9 and TLR2 signaling together account for MyD88-dependent control of parasitemia in Trypanosoma cruzi infection. *J. Immunol. Baltim. Md 1950* 177, 3515–3519. doi: 10.4049/jimmunol.177.6.3515

Bento, C. A., Melo, M. B., Previato, J. O., Mendonça-Previato, L., and Peçanha, L. M. (1996). Glycoinositolphospholipids purified from Trypanosoma cruzi stimulate Ig production in vitro. *J. Immunol. Baltim. Md 1950* 157, 4996–5001.

Bermejo, D. A., Amezcua Vesely, M. C., Khan, M., Acosta Rodríguez, E. V., Montes, C. L., Merino, M. C., et al. (2011). Trypanosoma cruzi infection induces a massive extrafollicular and follicular splenic B-cell response which is a high source of non-parasite-specific antibodies. *Immunology* 132, 123–133. doi: 10.1111/j.1365-2567.2010.03347.x

Bermejo, D. A., Jackson, S. W., Gorosito-Serran, M., Acosta-Rodriguez, E. V., Amezcua-Vesely, M. C., Sather, B. D., et al. (2013). Trypanosoma cruzi trans-sialidase initiates a program independent of the transcription factors RORγt and Ahr that leads to IL-17 production by activated B cells. *Nat. Immunol.* 14, 514–522. doi: 10.1038/ni.2569

Bott, E., Carneiro, A. B., Gimenez, G., López, M. G., Lammel, E. M., Atella, G. C., et al. (2018). Lipids From Trypanosoma cruzi Amastigotes of RA and K98 Strains Generate a Pro-inflammatory Response via TLR2/6. *Front. Cell. Infect. Microbiol.* 8, 151. doi: 10.3389/fcimb.2018.00151

Caetano, B. C., Carmo, B. B., Melo, M. B., Cerny, A., dos Santos, S. L., Bartholomeu, D. C., et al. (2011). Requirement of UNC93B1 reveals a critical role for TLR7 in host resistance to primary infection with Trypanosoma cruzi. *J. Immunol. Baltim. Md 1950* 187, 1903–1911. doi: 10.4049/jimmunol.1003911

Cai, C. W., Blase, J. R., Zhang, X., Eickhoff, C. S., and Hoft, D. F. (2016). Th17 Cells Are More Protective Than Th1 Cells Against the Intracellular Parasite Trypanosoma cruzi. *PLoS Pathog.* 12, e1005902. doi: 10.1371/journal.ppat.1005902

Campos, M. A., Closel, M., Valente, E. P., Cardoso, J. E., Akira, S., Alvarez-Leite, J. I., et al. (2004). Impaired production of proinflammatory cytokines and host resistance to acute infection with Trypanosoma cruzi in mice lacking functional myeloid differentiation factor 88. *J. Immunol. Baltim. Md 1950* 172, 1711–1718. doi: 10.4049/jimmunol.172.3.1711

Cardillo, F., de Pinho, R. T., Antas, P. R. Z., and Mengel, J. (2015). Immunity and immune modulation in Trypanosoma cruzi infection. *Pathog. Dis.* 73, ftv082. doi: 10.1093/femspd/ftv082

Cardillo, F., Voltarelli, J. C., Reed, S. G., and Silva, J. S. (1996). Regulation of Trypanosoma cruzi infection in mice by gamma interferon and interleukin 10: role of NK cells. *Infect. Immun.* 64, 128–134. doi: 10.1128/iai.64.1.128-134.1996

Cardoso, M. S., Reis-Cunha, J. L., and Bartholomeu, D. C. (2015). Evasion of the Immune Response by Trypanosoma cruzi during Acute Infection. *Front. Immunol.* 6, 659. doi: 10.3389/fimmu.2015.00659

De Arruda Hinds, L. B., Previato, L. M., Previato, J. O., Vos, Q., Mond, J. J., and Peçanha, L. M. (1999). Modulation of B-lymphocyte and NK cell activities by glycoinositolphospholipid purified from Trypanosoma cruzi. *Infect. Immun.* 67, 6177–6180. doi: 10.1128/IAI.67.11.6177-6180.1999

Flávia Nardy, A., Freire-de-Lima, C. G., and Morrot, A. (2015). Immune Evasion Strategies of Trypanosoma cruzi. *J. Immunol. Res.* 2015, 178947. doi: 10.1155/2015/178947

Fresno, M., and Gironès, N. (2018). Regulatory Lymphoid and Myeloid Cells Determine the Cardiac Immunopathogenesis of Trypanosoma cruzi Infection. *Front. Microbiol.* 9, 351. doi: 10.3389/fmicb.2018.00351

Gazzinelli, R. T., Pereira, M. E., Romanha, A., Gazzinelli, G., and Brener, Z. (1991). Direct lysis of Trypanosoma cruzi: a novel effector mechanism of protection mediated by human anti-gal antibodies. *Parasite Immunol.* 13, 345–356. doi: 10.1111/j.1365-3024.1991.tb00288.x

Gravina, H. D., Antonelli, L., Gazzinelli, R. T., and Ropert, C. (2013). Differential use of TLR2 and TLR9 in the regulation of immune responses during the infection with Trypanosoma cruzi. *PloS One* 8, e63100. doi: 10.1371/journal.pone.0063100

Kayama, H., and Takeda, K. (2010). The innate immune response to Trypanosoma cruzi infection. *Microbes Infect.* 12, 511–517. doi: 10.1016/j.micinf.2010.03.005

Koo, S.-J., Chowdhury, I. H., Szczesny, B., Wan, X., and Garg, N. J. (2016). Macrophages Promote Oxidative Metabolism To Drive Nitric Oxide Generation in Response to Trypanosoma cruzi. *Infect. Immun.* 84, 3527–3541. doi: 10.1128/IAI.00809-16

Krautz, G. M., Kissinger, J. C., and Krettli, A. U. (2000). The targets of the lytic antibody response against Trypanosoma cruzi. *Parasitol. Today Pers. Ed* 16, 31–34. doi: 10.1016/s0169-4758(99)01581-1

Kumar, S., and Tarleton, R. L. (1998). The relative contribution of antibody production and CD8+ T cell function to immune control of Trypanosoma cruzi. *Parasite Immunol.* 20, 207–216. doi: 10.1046/j.1365-3024.1998.00154.x

Ley, V., Andrews, N. W., Robbins, E. S., and Nussenzweig, V. (1988). Amastigotes of Trypanosoma cruzi sustain an infective cycle in mammalian cells. *J. Exp. Med.* 168, 649–659. doi: 10.1084/jem.168.2.649

Lidani, K. C. F., Andrade, F. A., Bavia, L., Damasceno, F. S., Beltrame, M. H., Messias-Reason, I. J., et al. (2019). Chagas Disease: From Discovery to a Worldwide Health Problem. *Front. Public Health* 7, 166. doi: 10.3389/fpubh.2019.00166

Lidani, K. C. F., Bavia, L., Ambrosio, A. R., and de Messias-Reason, I. J. (2017). The Complement System: A Prey of Trypanosoma cruzi. *Front. Microbiol.* 8, 607. doi: 10.3389/fmicb.2017.00607

Lieke, T., Graefe, S. E. B., Klauenberg, U., Fleischer, B., and Jacobs, T. (2004). NK cells contribute to the control of Trypanosoma cruzi infection by killing free parasites by perforin-independent mechanisms. *Infect. Immun.* 72, 6817–6825. doi: 10.1128/IAI.72.12.6817-6825.2004

Machado, F. S., Dutra, W. O., Esper, L., Gollob, K. J., Teixeira, M. M., Factor, S. M., et al. (2012). Current understanding of immunity to Trypanosoma cruzi infection and pathogenesis of Chagas disease. *Semin. Immunopathol.* 34, 753–770. doi: 10.1007/s00281-012-0351-7

Medeiros, M. M., Peixoto, J. R., Oliveira, A.-C., Cardilo-Reis, L., Koatz, V. L. G., Van Kaer, L., et al. (2007). Toll-like receptor 4 (TLR4)-dependent proinflammatory and immunomodulatory properties of the glycoinositolphospholipid (GIPL) from Trypanosoma cruzi. *J. Leukoc. Biol.* 82, 488–496. doi: 10.1189/jlb.0706478

Minoprio, P., Burlen, O., Pereira, P., Guilbert, B., Andrade, L., Hontebeyrie-Joskowicz, M., et al. (1988). Most B cells in acute Trypanosoma cruzi infection lack parasite specificity. *Scand. J. Immunol.* 28, 553–561. doi: 10.1111/j.1365-3083.1988.tb01487.x

Monteiro, A. C., Schmitz, V., Morrot, A., de Arruda, L. B., Nagajyothi, F., Granato, A., et al. (2007). Bradykinin B2 Receptors of dendritic cells, acting as sensors of kinins proteolytically released by Trypanosoma cruzi, are critical for the development of protective type-1 responses. *PLoS Pathog.* 3, e185. doi: 10.1371/journal.ppat.0030185

Monteon, V. (2019). Trypanosoma cruzi: the early contact between insect-derived metacyclic trypomastigotes and the mammalian cells. *Ann. Parasitol.* 65, 193–204. doi: 10.17420/ap6503.2019

Morrot, A., Villar, S. R., González, F. B., and Pérez, A. R. (2016). Evasion and Immuno-Endocrine Regulation in Parasite Infection: Two Sides of the Same Coin in Chagas Disease? *Front. Microbiol.* 7, 704. doi: 10.3389/fmicb.2016.00704

Muñoz-Fernández, M. A., Fernández, M. A., and Fresno, M. (1992). Activation of human macrophages for the killing of intracellular Trypanosoma cruzi by TNF-alpha and IFN-gamma through a nitric oxide-dependent mechanism. *Immunol. Lett.* 33, 35–40. doi: 10.1016/0165-2478(92)90090-b

Oliveira, A.-C., de Alencar, B. C., Tzelepis, F., Klezewsky, W., da Silva, R. N., Neves, F. S., et al. (2010). Impaired innate immunity in Tlr4(-/-) mice but preserved CD8+ T cell responses against Trypanosoma cruzi in Tlr4-, Tlr2-, Tlr9- or Myd88-deficient mice. *PLoS Pathog.* 6, e1000870. doi: 10.1371/journal.ppat.1000870

Oliveira, A.-C., Peixoto, J. R., de Arruda, L. B., Campos, M. A., Gazzinelli, R. T., Golenbock, D. T., et al. (2004). Expression of functional TLR4 confers proinflammatory responsiveness to Trypanosoma cruzi glycoinositolphospholipids and higher resistance to infection with T. cruzi. *J. Immunol. Baltim. Md 1950* 173, 5688–5696. doi: 10.4049/jimmunol.173.9.5688

Ouaissi, A., Guilvard, E., Delneste, Y., Caron, G., Magistrelli, G., Herbault, N., et al. (2002). The Trypanosoma cruzi Tc52-released protein induces human dendritic cell maturation, signals via Toll-like receptor 2, and confers protection against lethal infection. *J. Immunol. Baltim. Md 1950* 168, 6366–6374. doi: 10.4049/jimmunol.168.12.6366

Padilla, A. M., Simpson, L. J., and Tarleton, R. L. (2009). Insufficient TLR activation contributes to the slow development of CD8+ T cell responses in Trypanosoma cruzi infection. *J. Immunol. Baltim. Md 1950* 183, 1245–1252. doi: 10.4049/jimmunol.0901178

Poncini, C. V., and González-Cappa, S. M. (2017). Dual role of monocyte-derived dendritic cells in Trypanosoma cruzi infection. *Eur. J. Immunol.* 47, 1936–1948. doi: 10.1002/eji.201646830

Rodríguez, A., Samoff, E., Rioult, M. G., Chung, A., and Andrews, N. W. (1996). Host cell invasion by trypanosomes requires lysosomes and microtubule/kinesin-mediated transport. *J. Cell Biol.* 134, 349–362. doi: 10.1083/jcb.134.2.349

Rodriguez, T., Pacheco-Fernández, T., Vázquez-Mendoza, A., Nieto-Yañez, O., Juárez-Avelar, I., Reyes, J. L., et al. (2020). MGL1 Receptor Plays a Key Role in the Control of T. cruzi Infection by Increasing Macrophage Activation through Modulation of ERK1/2, c-Jun, NF-κB and NLRP3 Pathways. *Cells* 9, 108. doi: 10.3390/cells9010108

Sardinha, L. R., Elias, R. M., Mosca, T., Bastos, K. R. B., Marinho, C. R. F., D’Império Lima, M. R., et al. (2006). Contribution of NK, NK T, gamma delta T, and alpha beta T cells to the gamma interferon response required for liver protection against Trypanosoma cruzi. *Infect. Immun.* 74, 2031–2042. doi: 10.1128/IAI.74.4.2031-2042.2006
