# Supplementary material for: Towards a map of the immune system manipulation network by Trypanosoma cruzi
Source: Front Cell Infect Microbiol. 2026 Jan 6;15:1711520. doi: 10.3389/fcimb.2025.1711520 (PMC12816262; doi:10.3389/fcimb.2025.1711520)
Supplement: Supplementary file 2 [file Table2.docx]

**Table II Parasite Components Involved in Host Immune System Subversion**

| **Component** |  | **Description** | **Reference** | **Used as vaccine candidate** |
| --- | --- | --- | --- | --- |
| **Trans-sialidase (TS) super family** | Include active and inactive TS members | Trans-sialidase/trans-sialidase-like (TS) proteins constitute a large polymorphic superfamily comprising more than 1400 genes that have been divided into 8 groups. The first group includes active TS found in T. cruzi. Since the parasite is unable to synthesize sialic acid, this enzyme cleaves sialic acid from host glycoconjugates and transfers it to the parasite, which becomes highly sialylated in mucin-like structures. Several immunomodulatory functions have been described for active TS. The second group includes proteins involved in host invasion, while the third encompasses proteins linked to complement evasion. The remaining groups contain proteins of unknown function. Collectively, the extensive repertoire of antigenic peptides within this family may enable the parasite to distract and evade host immune responses. | (Freitas et al., 2011; Chiurillo et al., 2016; Nardy et al., 2016) | **Yes.** (Giddings et al., 2010; Bontempi et al., 2015, 2017; Freire-de-Lima et al., 2015; Prochetto et al., 2017; Dumonteil and Herrera, 2021; Gamba et al., 2021, 2025; Castro et al., 2023; Borgna et al., 2024; Dos Santos et al., 2025) |
| **Polyclonal B cell antigens** | Proline racemase | Proline racemases (PR) are enzymes that interconvert free L- and D-proline enantiomers. A 45kDa PR is only expressed and released by infective metacyclic and bloodstream forms of the parasite. It has been described that PR is an efficient mitogen for B lymphocytes and is significantly implicated in non-specific polyclonal activation of lymphoid cells, preventing the development of effective parasite-directed immune responses. | (Reina-San-Martín et al., 2000) | **Yes**. (Bryan and Norris, 2010) |
|  | Tc24 | Tc24 has been shown to elicit a nonspecific and T-independent B cell response, secreting mainly IgM both in vivo and in vitro. | (Da Silva et al., 1998) | **Yes.** (Martinez-Campos et al., 2015; Dumonteil and Herrera, 2021) |
|  | Shed acute phase antigen (SAPA, from TS) | Shed acute phase antigen is a fragment of trans-sialidase located in the most carboxy-terminal part of the protein from many group I members. It has been described that SAPA can activate B cells in a T cell-independent manner, leading to polyclonal antibody secretion. | (Gao et al., 2002) | **Yes.**  (Dos Santos et al., 2025) |
| **Complement regulatory proteins** | Calreticulin | It is a protein able to bind to the collagen-like domains of MBL, ficolin-2 and C1q, affecting the lectin and classical pathways of complement activation | (Ramírez et al., 2011; Lidani et al., 2017; Ramírez-Toloza and Ferreira, 2017) | **Yes.** (Sánchez-Valdéz et al., 2015) |
|  | Tc-DAF | It is a protein that shares similarity with a host decay accelerating factor that can bind to C3b and C4b accelerating the dissociation of C3 convertase. This is an event that would affect the classical, alternative, and eventually the lectin pathways of complement activation. | (Lidani et al., 2017) | **No.** |
|  | TcCRP | It is a protein that belongs to the inactive trans-sialidase family (group III) and is glycosylphosphatidylinositol (GPI)-anchored to the cell membrane of the trypomastigote. It is able to bind to C3b and C4b in a way that impairs the function of C3 convertase, also affecting the classical, alternative, and eventually the lectin pathway of complement activation. | (Lidani et al., 2017) | **Yes.** (Sepulveda et al., 2000) |
|  | TcCRIT | It is a protein that binds to C2 and prevents its cleavage by C1s and MASP-2, interfering with the classical and lectin pathways. | (Lidani et al., 2017) | **No.** |
|  | gp58/68 | It has been reported that gp58/68 is an analog of a host regulatory protein that interacts with Factor B, blocking its binding to C3b and influencing the alternative pathway. | (Lidani et al., 2017) | **No.** |
| **Cruzipain** |  | Cruzipain has been described as a highly immunogenic protein of about 52–58 kDa. It is a major cysteine protease that has been implicated in several pathways of host immune manipulation. | (Giordanengo et al., 2002; Doyle et al., 2011) | **Yes**. (Cazorla et al., 2008; Cerny et al., 2020; Pinazo et al., 2024) |
| **GIPLs** |  | GIPLs are glycolipids composed of a glycan linked to a lipid anchor. They are among the most abundant cell surface molecules on *T. cruzi*. | (Brodskyn et al., 2002; DosReis et al., 2002) | **No.** |
| **Bioactive lipids** | Thromboxane A2 | It has been reported that Thromboxane A2 from the parasite may influence the host immune response. | (Ashton et al., 2007; de Almeida et al., 2018) | **No**. |
|  | Prostaglandin E2 (PGE2) | PGE2 from lipid bodies may affect macrophages. | (de Almeida et al., 2018) | **No.** |
| **SSP4** |  | It has been postulated that this protein has immunomodulatory properties. | (Flores-García et al., 2011; Morán-Utrera et al., 2012) | **Yes.**  (Arce-Fonseca et al., 2011, 2015; Rodríguez-Morales et al., 2012) |
| **Tc52** |  | It is a glutathione disulfide thioltransferase that has been involved in several pathways related to immunomodulation. | (Moutiez et al., 1997; Ouaissi et al., 2002) | **Yes**. (Ouaissi et al., 2002; Matos et al., 2017; Vázquez et al., 2024) |
| **EVs** |  | *T. cruzi* releases extracellular vesicles (EVs), carrying proteins related to metabolism, signaling, survival, and parasite virulence. After EV endocytosis, these vesicles may reach the cytoplasm and release their contents. | (Bayer-Santos et al., 2013; Lovo-Martins et al., 2018) | **Yes.** (Aggio et al., 2025) |
| **Lipid bodies** |  | Lipid bodies (LBs) are lipid rich organelles. PGE2 from *T. cruzi* LBs may contribute to impairing macrophage Th1 polarization. | (de Almeida et al., 2018) | No. |
| **AgC10** |  | AgC10 is a *T. cruzi* mucin expressed in epimastigotes and amastigotes and has been reported to block IL-2 synthesis, playing a role in T-cell immunosuppression. | (Alcaide and Fresno, 2004) | **No.** |
| **TcRpL7** |  | It is a repetitive fragment of the ribosomal protein L7a that may suppress B-cell proliferation. | (Toro Acevedo et al., 2017) | **No.** |
| **P21** |  | It is a protein that has been related to the evasion mechanisms that enable parasite persistence with low or undetectable parasitemia. | (da Silva et al., 2009; Teixeira et al., 2019) | **Yes.** (Pérez Brandán et al., 2019) |
| **MASP (Mucin Associated Surface Proteins)** |  | The large repertoire of antigenic peptides included in the family may be used by the parasite to distract and evade the host immune response. | (dos Santos et al., 2012) | **Yes.** (Serna et al., 2014) |

Aggio, J. B., Vedam, V. V., Nisimura, L. M., da Silva, R. V., Lovo-Martins, M. I., Borges, B. S., et al. (2025). Trypanosomatid Extracellular Vesicles as Potential Immunogens for Chagas Disease. *Int. J. Mol. Sci.* 26, 1544. doi: 10.3390/ijms26041544

Alcaide, P., and Fresno, M. (2004). The Trypanosoma cruzi membrane mucin AgC10 inhibits T cell activation and IL-2 transcription through L-selectin. *Int. Immunol.* 16, 1365–1375. doi: 10.1093/intimm/dxh138

Arce-Fonseca, M., Ramos-Ligonio, A., López-Monteón, A., Salgado-Jiménez, B., Talamás-Rohana, P., and Rosales-Encina, J. L. (2011). A DNA vaccine encoding for TcSSP4 induces protection against acute and chronic infection in experimental Chagas disease. *Int. J. Biol. Sci.* 7, 1230–1238. doi: 10.7150/ijbs.7.1230

Arce-Fonseca, M., Rios-Castro, M., Carrillo-Sánchez, S. del C., Martínez-Cruz, M., and Rodríguez-Morales, O. (2015). Prophylactic and therapeutic DNA vaccines against Chagas disease. *Parasit. Vectors* 8, 121. doi: 10.1186/s13071-015-0738-0

Ashton, A. W., Mukherjee, S., Nagajyothi, F. N. U., Huang, H., Braunstein, V. L., Desruisseaux, M. S., et al. (2007). Thromboxane A2 is a key regulator of pathogenesis during Trypanosoma cruzi infection. *J. Exp. Med.* 204, 929–940. doi: 10.1084/jem.20062432

Bayer-Santos, E., Aguilar-Bonavides, C., Rodrigues, S. P., Cordero, E. M., Marques, A. F., Varela-Ramirez, A., et al. (2013). Proteomic analysis of Trypanosoma cruzi secretome: characterization of two populations of extracellular vesicles and soluble proteins. *J. Proteome Res.* 12, 883–897. doi: 10.1021/pr300947g

Bontempi, I. A., Vicco, M. H., Cabrera, G., Villar, S. R., González, F. B., Roggero, E. A., et al. (2015). Efficacy of a trans-sialidase-ISCOMATRIX subunit vaccine candidate to protect against experimental Chagas disease. *Vaccine* 33, 1274–1283. doi: 10.1016/j.vaccine.2015.01.044

Bontempi, I., Fleitas, P., Poato, A., Vicco, M., Rodeles, L., Prochetto, E., et al. (2017). Trans-sialidase overcomes many antigens to be used as a vaccine candidate against Trypanosoma cruzi. *Immunotherapy* 9, 555–565. doi: 10.2217/imt-2017-0009

Borgna, E., Prochetto, E., Gamba, J. C., Vermeulen, E. M., Poncini, C. V., Cribb, P., et al. (2024). Control of myeloid-derived suppressor cell dynamics potentiates vaccine protection in multiple mouse models of Trypanosoma cruzi infection. *Front. Immunol.* 15, 1484290. doi: 10.3389/fimmu.2024.1484290

Brodskyn, C., Patricio, J., Oliveira, R., Lobo, L., Arnholdt, A., Mendonça-Previato, L., et al. (2002). Glycoinositolphospholipids from Trypanosoma cruzi interfere with macrophages and dendritic cell responses. *Infect. Immun.* 70, 3736–3743. doi: 10.1128/IAI.70.7.3736-3743.2002

Bryan, M. A., and Norris, K. A. (2010). Genetic immunization converts the trypanosoma cruzi B-Cell mitogen proline racemase to an effective immunogen. *Infect. Immun.* 78, 810–822. doi: 10.1128/IAI.00926-09

Castro, J. T., Brito, R., Hojo-Souza, N. S., Azevedo, B., Salazar, N., Ferreira, C. P., et al. (2023). ASP-2/Trans-sialidase chimeric protein induces robust protective immunity in experimental models of Chagas’ disease. *NPJ Vaccines* 8, 81. doi: 10.1038/s41541-023-00676-0

Cazorla, S. I., Becker, P. D., Frank, F. M., Ebensen, T., Sartori, M. J., Corral, R. S., et al. (2008). Oral vaccination with Salmonella enterica as a cruzipain-DNA delivery system confers protective immunity against Trypanosoma cruzi. *Infect. Immun.* 76, 324–333. doi: 10.1128/IAI.01163-07

Cerny, N., Bivona, A. E., Sanchez Alberti, A., Trinitario, S. N., Morales, C., Cardoso Landaburu, A., et al. (2020). Cruzipain and Its Physiological Inhibitor, Chagasin, as a DNA-Based Therapeutic Vaccine Against Trypanosoma cruzi. *Front. Immunol.* 11, 565142. doi: 10.3389/fimmu.2020.565142

Chiurillo, M. A., Cortez, D. R., Lima, F. M., Cortez, C., Ramírez, J. L., Martins, A. G., et al. (2016). The diversity and expansion of the trans-sialidase gene family is a common feature in Trypanosoma cruzi clade members. *Infect. Genet. Evol. J. Mol. Epidemiol. Evol. Genet. Infect. Dis.* 37, 266–274. doi: 10.1016/j.meegid.2015.11.024

Da Silva, A. C., Espinoza, A. G., Taibi, A., Ouaissi, A., and Minoprio, P. (1998). A 24,000 MW Trypanosoma cruzi antigen is a B-cell activator. *Immunology* 94, 189–196. doi: 10.1046/j.1365-2567.1998.00498.x

da Silva, C. V., Kawashita, S. Y., Probst, C. M., Dallagiovanna, B., Cruz, M. C., da Silva, E. A., et al. (2009). Characterization of a 21kDa protein from Trypanosoma cruzi associated with mammalian cell invasion. *Microbes Infect.* 11, 563–570. doi: 10.1016/j.micinf.2009.03.007

de Almeida, P. E., Toledo, D. A. M., Rodrigues, G. S. C., and D’Avila, H. (2018). Lipid Bodies as Sites of Prostaglandin E2 Synthesis During Chagas Disease: Impact in the Parasite Escape Mechanism. *Front. Microbiol.* 9, 499. doi: 10.3389/fmicb.2018.00499

Dos Santos, N. S. A., de Almeida-Júnior, C. R., Ricci, M. F., Sanches, R. C. O., Fernandes, R. S., Burle-Caldas, G. de A., et al. (2025). RNA and protein immunization with Trypanosoma cruzi trans-sialidase containing SAPA repeats protects mice against infection and promotes a balanced inflammatory response. *Front. Cell. Infect. Microbiol.* 15, 1681807. doi: 10.3389/fcimb.2025.1681807

dos Santos, S. L., Freitas, L. M., Lobo, F. P., Rodrigues-Luiz, G. F., Mendes, T. A. de O., Oliveira, A. C. S., et al. (2012). The MASP family of Trypanosoma cruzi: changes in gene expression and antigenic profile during the acute phase of experimental infection. *PLoS Negl. Trop. Dis.* 6, e1779. doi: 10.1371/journal.pntd.0001779

DosReis, G. A., Peçanha, L. M. T., Bellio, M., Previato, J. O., and Mendonça-Previato, L. (2002). Glycoinositol phospholipids from Trypanosoma cruzi transmit signals to the cells of the host immune system through both ceramide and glycan chains. *Microbes Infect.* 4, 1007–1013. doi: 10.1016/s1286-4579(02)01616-7

Doyle, P. S., Zhou, Y. M., Hsieh, I., Greenbaum, D. C., McKerrow, J. H., and Engel, J. C. (2011). The Trypanosoma cruzi protease cruzain mediates immune evasion. *PLoS Pathog.* 7, e1002139. doi: 10.1371/journal.ppat.1002139

Dumonteil, E., and Herrera, C. (2021). The Case for the Development of a Chagas Disease Vaccine: Why? How? When? *Trop. Med. Infect. Dis.* 6, 16. doi: 10.3390/tropicalmed6010016

Flores-García, Y., Rosales-Encina, J. L., Satoskar, A. R., and Talamás-Rohana, P. (2011). IL-10-IFN-γ double producers CD4+ T cells are induced by immunization with an amastigote stage specific derived recombinant protein of Trypanosoma cruzi. *Int. J. Biol. Sci.* 7, 1093–1100. doi: 10.7150/ijbs.7.1093

Freire-de-Lima, L., Fonseca, L. M., Oeltmann, T., Mendonça-Previato, L., and Previato, J. O. (2015). The trans-sialidase, the major Trypanosoma cruzi virulence factor: Three decades of studies. *Glycobiology* 25, 1142–1149. doi: 10.1093/glycob/cwv057

Freitas, L. M., dos Santos, S. L., Rodrigues-Luiz, G. F., Mendes, T. A. O., Rodrigues, T. S., Gazzinelli, R. T., et al. (2011). Genomic analyses, gene expression and antigenic profile of the trans-sialidase superfamily of Trypanosoma cruzi reveal an undetected level of complexity. *PloS One* 6, e25914. doi: 10.1371/journal.pone.0025914

Gamba, J. C., Borgna, E., Prochetto, E., Pérez, A. R., Batista-Duharte, A., Marcipar, I., et al. (2025). Integrating Cellular Immune Biomarkers with Machine Learning to Identify Potential Correlates of Protection for a Trypanosoma cruzi Vaccine. *Vaccines* 13, 915. doi: 10.3390/vaccines13090915

Gamba, J. C., Roldán, C., Prochetto, E., Lupi, G., Bontempi, I., Poncini, C. V., et al. (2021). Targeting Myeloid-Derived Suppressor Cells to Enhance a Trans-Sialidase-Based Vaccine Against Trypanosoma cruzi. *Front. Cell. Infect. Microbiol.* 11, 671104. doi: 10.3389/fcimb.2021.671104

Gao, W., Wortis, H. H., and Pereira, M. A. (2002). The Trypanosoma cruzi trans-sialidase is a T cell-independent B cell mitogen and an inducer of non-specific Ig secretion. *Int. Immunol.* 14, 299–308. doi: 10.1093/intimm/14.3.299

Giddings, O. K., Eickhoff, C. S., Sullivan, N. L., and Hoft, D. F. (2010). Intranasal vaccinations with the trans-sialidase antigen plus CpG Adjuvant induce mucosal immunity protective against conjunctival Trypanosoma cruzi challenges. *Infect. Immun.* 78, 1333–1338. doi: 10.1128/IAI.00278-09

Giordanengo, L., Guiñazú, N., Stempin, C., Fretes, R., Cerbán, F., and Gea, S. (2002). Cruzipain, a major Trypanosoma cruzi antigen, conditions the host immune response in favor of parasite. *Eur. J. Immunol.* 32, 1003–1011. doi: 10.1002/1521-4141(200204)32:4<1003::AID-IMMU1003>3.0.CO;2-P

Lidani, K. C. F., Bavia, L., Ambrosio, A. R., and de Messias-Reason, I. J. (2017). The Complement System: A Prey of Trypanosoma cruzi. *Front. Microbiol.* 8, 607. doi: 10.3389/fmicb.2017.00607

Lovo-Martins, M. I., Malvezi, A. D., Zanluqui, N. G., Lucchetti, B. F. C., Tatakihara, V. L. H., Mörking, P. A., et al. (2018). Extracellular Vesicles Shed By Trypanosoma cruzi Potentiate Infection and Elicit Lipid Body Formation and PGE2 Production in Murine Macrophages. *Front. Immunol.* 9, 896. doi: 10.3389/fimmu.2018.00896

Martinez-Campos, V., Martinez-Vega, P., Ramirez-Sierra, M. J., Rosado-Vallado, M., Seid, C. A., Hudspeth, E. M., et al. (2015). Expression, purification, immunogenicity, and protective efficacy of a recombinant Tc24 antigen as a vaccine against Trypanosoma cruzi infection in mice. *Vaccine* 33, 4505–4512. doi: 10.1016/j.vaccine.2015.07.017

Matos, M. N., Cazorla, S. I., Schulze, K., Ebensen, T., Guzmán, C. A., and Malchiodi, E. L. (2017). Immunization with Tc52 or its amino terminal domain adjuvanted with c-di-AMP induces Th17+Th1 specific immune responses and confers protection against Trypanosoma cruzi. *PLoS Negl. Trop. Dis.* 11, e0005300. doi: 10.1371/journal.pntd.0005300

Morán-Utrera, Y., López-Monteon, A., Rosales-Encina, J. L., Méndez-Bolaina, E., and Ramos-Ligonio, A. (2012). Trypanosoma cruzi SSP4 Amastigote Protein Induces Expression of Immunoregulatory and Immunosuppressive Molecules in Peripheral Blood Mononuclear Cells. *J. Trop. Med.* 2012, 829139. doi: 10.1155/2012/829139

Moutiez, M., Quéméneur, E., Sergheraert, C., Lucas, V., Tartar, A., and Davioud-Charvet, E. (1997). Glutathione-dependent activities of Trypanosoma cruzi p52 makes it a new member of the thiol:disulphide oxidoreductase family. *Biochem. J.* 322 ( Pt 1), 43–48. doi: 10.1042/bj3220043

Nardy, A. F. F. R., Freire-de-Lima, C. G., Pérez, A. R., and Morrot, A. (2016). Role of Trypanosoma cruzi Trans-sialidase on the Escape from Host Immune Surveillance. *Front. Microbiol.* 7, 348. doi: 10.3389/fmicb.2016.00348

Ouaissi, A., Guilvard, E., Delneste, Y., Caron, G., Magistrelli, G., Herbault, N., et al. (2002). The Trypanosoma cruzi Tc52-released protein induces human dendritic cell maturation, signals via Toll-like receptor 2, and confers protection against lethal infection. *J. Immunol. Baltim. Md 1950* 168, 6366–6374. doi: 10.4049/jimmunol.168.12.6366

Pérez Brandán, C., Mesias, A. C., Acuña, L., Teixeira, T. L., and da Silva, C. V. (2019). Evaluation of pathogen P21 protein as a potential modulator of the protective immunity induced by Trypanosoma cruzi attenuated parasites. *Mem. Inst. Oswaldo Cruz* 114, e180571. doi: 10.1590/0074-02760180571

Pinazo, M. J., Malchiodi, E., Ioset, J.-R., Bivona, A., Gollob, K. J., and Dutra, W. O. (2024). Challenges and advancements in the development of vaccines and therapies against Chagas disease. *Lancet Microbe* 5, 100972. doi: 10.1016/j.lanmic.2024.100972

Prochetto, E., Roldán, C., Bontempi, I. A., Bertona, D., Peverengo, L., Vicco, M. H., et al. (2017). Trans-sialidase-based vaccine candidate protects against Trypanosoma cruzi infection, not only inducing an effector immune response but also affecting cells with regulatory/suppressor phenotype. *Oncotarget* 8, 58003–58020. doi: 10.18632/oncotarget.18217

Ramírez, G., Valck, C., Molina, M. C., Ribeiro, C. H., López, N., Sánchez, G., et al. (2011). Trypanosoma cruzi calreticulin: a novel virulence factor that binds complement C1 on the parasite surface and promotes infectivity. *Immunobiology* 216, 265–273. doi: 10.1016/j.imbio.2010.04.001

Ramírez-Toloza, G., and Ferreira, A. (2017). Trypanosoma cruzi Evades the Complement System as an Efficient Strategy to Survive in the Mammalian Host: The Specific Roles of Host/Parasite Molecules and Trypanosoma cruzi Calreticulin. *Front. Microbiol.* 8, 1667. doi: 10.3389/fmicb.2017.01667

Reina-San-Martín, B., Degrave, W., Rougeot, C., Cosson, A., Chamond, N., Cordeiro-Da-Silva, A., et al. (2000). A B-cell mitogen from a pathogenic trypanosome is a eukaryotic proline racemase. *Nat. Med.* 6, 890–897. doi: 10.1038/78651

Rodríguez-Morales, O., Pérez-Leyva, M. M., Ballinas-Verdugo, M. A., Carrillo-Sánchez, S. C., Rosales-Encina, J. L., Alejandre-Aguilar, R., et al. (2012). Plasmid DNA immunization with Trypanosoma cruzi genes induces cardiac and clinical protection against Chagas disease in the canine model. *Vet. Res.* 43, 79. doi: 10.1186/1297-9716-43-79

Sánchez-Valdéz, F. J., Pérez Brandán, C., Ferreira, A., and Basombrío, M. Á. (2015). Gene-deleted live-attenuated Trypanosoma cruzi parasites as vaccines to protect against Chagas disease. *Expert Rev. Vaccines* 14, 681–697. doi: 10.1586/14760584.2015.989989

Sepulveda, P., Hontebeyrie, M., Liegeard, P., Mascilli, A., and Norris, K. A. (2000). DNA-Based immunization with Trypanosoma cruzi complement regulatory protein elicits complement lytic antibodies and confers protection against Trypanosoma cruzi infection. *Infect. Immun.* 68, 4986–4991. doi: 10.1128/IAI.68.9.4986-4991.2000

Serna, C., Lara, J. A., Rodrigues, S. P., Marques, A. F., Almeida, I. C., and Maldonado, R. A. (2014). A synthetic peptide from Trypanosoma cruzi mucin-like associated surface protein as candidate for a vaccine against Chagas disease. *Vaccine* 32, 3525–3532. doi: 10.1016/j.vaccine.2014.04.026

Teixeira, T. L., Castilhos, P., Rodrigues, C. C., da Silva, A. A., Brígido, R. T., Teixeira, S. C., et al. (2019). Experimental evidences that P21 protein controls Trypanosoma cruzi replication and modulates the pathogenesis of infection. *Microb. Pathog.* 135, 103618. doi: 10.1016/j.micpath.2019.103618

Toro Acevedo, C. A., Valente, B. M., Burle-Caldas, G. A., Galvão-Filho, B., Santiago, H. da C., Esteves Arantes, R. M., et al. (2017). Down Modulation of Host Immune Response by Amino Acid Repeats Present in a Trypanosoma cruzi Ribosomal Antigen. *Front. Microbiol.* 8, 2188. doi: 10.3389/fmicb.2017.02188

Vázquez, M. E., Zabala, B. A., Mesías, A. C., Biscari, L., Kaufman, C. D., Alloatti, A., et al. (2024). Protective Efficacy of the Epitope-Conjugated Antigen N-Tc52/TSkb20 in Mitigating Trypanosoma cruzi Infection through CD8+ T-Cells and IFNγ Responses. *Vaccines* 12, 621. doi: 10.3390/vaccines12060621
