# Supplementary material for: Towards a map of the immune system manipulation network by Trypanosoma cruzi
Source: Front Cell Infect Microbiol. 2026 Jan 6;15:1711520. doi: 10.3389/fcimb.2025.1711520 (PMC12816262; doi:10.3389/fcimb.2025.1711520)
Supplement: Supplementary file 3 [file Table3.docx]

**Supplementary Table III.**

|  | **Basic description of relevant host immune components during *T. cruzi* infection** |
| --- | --- |
| **Macrophages** | Macrophages are widely distributed in tissues and play a pivotal role in the early immune response. These cells can eliminate pathogens and elicit immune responses, but under certain conditions they may also serve as a niche that permits pathogen survival and replication (Romano et al., 2012; Cardoso et al., 2015; Acevedo et al., 2018). In general terms, stimulated macrophages can be polarized into M1, which are associated with a Th1-type response and the production of IL-12 and IFN-γ, or into M2, which are associated with a Th2-type response and the production of IL-10 and TGF-β. The balance between these polarization states is critical in determining the outcome of the immune response (Cerbán et al., 2020). |
| **Dendritic cells** | DCs are considered sentinels of the immune system. In fact, these cells connect the innate and adaptive immune system; DCs are present in most non-lymphoid tissues and, after the antigen (Ag) encounter, traffic to lymphoid organs for Ag presentation. DCs shape adaptive immune responses and regulate T cell commitment, differentiation, and function (Merad et al., 2013). Over decades of research, it has become clear that DCs comprise different cell subsets with particular development and functions, arising from a specific hematopoietic progenitor (Naik et al., 2007).  Two major ontogenetically different DC populations have been described: plasmacytoid (pDCs) and conventional (cDCs). They share a common progenitor in bone marrow that gives rise to different precursors with different life cycles, localization, and marker expression, including MHCII and pattern recognition receptors (PRRs)—factors that influence the function and properties of these cells (Vu Manh et al., 2015). Subsets are defined not only on phenotypic differences but also on developmental specificity and functional specialization (Randolph et al., 2008; Satpathy et al., 2012; Vu Manh et al., 2015). |
| **Complement system** | Under normal conditions, the complement can label, opsonize, and destroy pathogens, and also plays a role in coordinating inflammation and interacting with the adaptive immune system (Ermert et al., 2019). The complement functions as a cascade of proteolytic events that can be activated by three pathways: classical (CPath), lectin (LPath), and alternative (APath), all of which converge in the formation of a C3 convertase complex (Lidani et al., 2017; Acevedo et al., 2018).  The CPath is typically activated when the C1 complex binds antigen-antibody complexes, or in some cases, directly to PAMPs. This interaction drives cleavage of C4 and C2, forming the C3 convertase (C4b-2a). The LPath is triggered when microbial carbohydrates are recognized by mannose-binding lectin (MBL) or other lectins, which then activate MBL-associated serine proteases (MASPs) that also cleave C4 and C2, generating a C3 convertase as in the CPath. By contrast, the APath relies on the spontaneous hydrolysis of C3, which recruits factor B (FB). Cleavage of FB by factor D (FD) generates the alternative C3 convertase (C3b-Bb) (Ermert et al., 2019).  Once formed, C3 convertases bound to target membranes are able to activate C3, releasing C3a and incorporating C3b into the C5 convertase complex (C4b-2a-3b for CPath/LPath or C3b-Bb-3b for APath). The C5 convertase then cleaves C5, producing the potent inflammatory mediator C5a, while initiating assembly of the terminal complement complex. C5b sequentially binds C6 and C7, C8, and multiple C9 molecules to form the membrane attack complex (MAC), a ring-like pore structure that disrupts membranes and ultimately causes lysis (Ramírez-Toloza and Ferreira, 2017). |
| **Neutrophils and Monocytes** | Neutrophils are frontline myeloid effector cells of the innate immune response involved in the activation, regulation, and effector functions of both innate and adaptive immune cells (Brandau et al., 2013; Zhou et al., 2018). They are the predominant leukocyte subset in human blood and one of the major subsets in mice. To recognize, take up, and eliminate pathogens, neutrophils are equipped with numerous antimicrobial mediators, such as myeloperoxidase (MPO), neutrophil elastase (NE), defensins, cathelicidins, and matrix metalloproteinases (MMPs), many of which are preformed and stored in specialized granules (Brandau et al., 2013; Zhou et al., 2018).  Neutrophil development occurs in the BM and involves several defined steps that can be observed at a histological level: neutrophils progressively condense their nucleus from a toroidal shape in pre-neutrophil cells to a poly-segmented shape in BM-mature and blood neutrophils (Evrard et al., 2018).  In a healthy state, normal myelopoiesis ensures constant replenishment of circulating neutrophils (CD11b+Ly6G+CXCR2+), which have a short life-span of ~ 19 hours in circulation (Evrard et al., 2018).  Monocytes, another myeloid subset, originate in BM and are characterized by their high plasticity. As innate immune cells, monocytes are phagocytes, produce oxygen reactive species, and participate in cellular processes including tissue repair and regeneration during heart diseases (Gómez-Olarte et al., 2019).  They circulate in the blood for a few days before infiltrating different tissues, where they differentiate into macrophages or DCs (Gonzalez-Mejia and Doseff, 2009; Canè et al., 2019). The most common classification divides blood monocytes into the following groups: inflammatory monocytes (iMO; in humans classical CD14^++^ CD16^–^; in mice Ly6C^+^), intermediate (in humans CD14^++^ CD16^+^), and non-classical (in humans CD14^+^ CD16^++^ ; in mice Ly6C^low/-^) (Mildner et al., 2016; Gómez-Olarte et al., 2019). During pathological processes, blood monocytes can produce cytokines, clear pathogens, present antigens, and participate in wound healing and responses against tumors (Canè et al., 2019). |
| **HPA (hypothalamus–pituitary–adrenal) axis** | Under physiological conditions, immune, neural, and endocrine networks operate as an integrated system that detects danger signals associated with pathogen invasion or organ damage and subsequently activates defense mechanisms (Blalock, 2005; Del Rey and Besedovsky, 2017). In mammals, pathogen threats elicit acute, short-term stress responses. The integration of these stress signals is primarily orchestrated by the hypothalamus–pituitary–adrenal (HPA) axis, leading to a transient increase in glucocorticoid levels (Dhabhar, 2009). This increase supports the activation of innate defense mechanisms. However, when the stress response is prolonged, it can become detrimental to the host. Indeed, the same components involved in initiating and regulating inflammation -such as anti-inflammatory cytokines, glucocorticoids, and regulatory T cells- may also mediate harmful effects (McEwen, 1998). |

Acevedo, G. R., Girard, M. C., and Gómez, K. A. (2018). The Unsolved Jigsaw Puzzle of the Immune Response in Chagas Disease. *Front Immunol* 9, 1929. doi: 10.3389/fimmu.2018.01929

Blalock, J. E. (2005). The immune system as the sixth sense. *J Intern Med* 257, 126–138. doi: 10.1111/j.1365-2796.2004.01441.x

Brandau, S., Dumitru, C. A., and Lang, S. (2013). Protumor and antitumor functions of neutrophil granulocytes. *Semin Immunopathol* 35, 163–176. doi: 10.1007/s00281-012-0344-6

Canè, S., Ugel, S., Trovato, R., Marigo, I., De Sanctis, F., Sartoris, S., et al. (2019). The Endless Saga of Monocyte Diversity. *Front Immunol* 10, 1786. doi: 10.3389/fimmu.2019.01786

Cardoso, M. S., Reis-Cunha, J. L., and Bartholomeu, D. C. (2015). Evasion of the Immune Response by Trypanosoma cruzi during Acute Infection. *Front Immunol* 6, 659. doi: 10.3389/fimmu.2015.00659

Cerbán, F. M., Stempin, C. C., Volpini, X., Carrera Silva, E. A., Gea, S., and Motran, C. C. (2020). Signaling pathways that regulate Trypanosoma cruzi infection and immune response. *Biochim Biophys Acta Mol Basis Dis* 1866, 165707. doi: 10.1016/j.bbadis.2020.165707

Del Rey, A., and Besedovsky, H. O. (2017). Immune-Neuro-Endocrine Reflexes, Circuits, and Networks: Physiologic and Evolutionary Implications. *Front Horm Res* 48, 1–18. doi: 10.1159/000452902

Dhabhar, F. S. (2009). A hassle a day may keep the pathogens away: The fight-or-flight stress response and the augmentation of immune function. *Integr Comp Biol* 49, 215–236. doi: 10.1093/icb/icp045

Ermert, D., Ram, S., and Laabei, M. (2019). The hijackers guide to escaping complement: Lessons learned from pathogens. *Mol Immunol* 114, 49–61. doi: 10.1016/j.molimm.2019.07.018

Evrard, M., Kwok, I. W. H., Chong, S. Z., Teng, K. W. W., Becht, E., Chen, J., et al. (2018). Developmental Analysis of Bone Marrow Neutrophils Reveals Populations Specialized in Expansion, Trafficking, and Effector Functions. *Immunity* 48, 364-379.e8. doi: 10.1016/j.immuni.2018.02.002

Gómez-Olarte, S., Bolaños, N. I., Echeverry, M., Rodríguez, A. N., Cuéllar, A., Puerta, C. J., et al. (2019). Intermediate Monocytes and Cytokine Production Associated With Severe Forms of Chagas Disease. *Front Immunol* 10, 1671. doi: 10.3389/fimmu.2019.01671

Gonzalez-Mejia, M. E., and Doseff, A. I. (2009). Regulation of monocytes and macrophages cell fate. *Front Biosci (Landmark Ed)* 14, 2413–2431. doi: 10.2741/3387

Lidani, K. C. F., Bavia, L., Ambrosio, A. R., and de Messias-Reason, I. J. (2017). The Complement System: A Prey of Trypanosoma cruzi. *Front Microbiol* 8, 607. doi: 10.3389/fmicb.2017.00607

McEwen, B. S. (1998). Protective and damaging effects of stress mediators. *N Engl J Med* 338, 171–179. doi: 10.1056/NEJM199801153380307

Merad, M., Sathe, P., Helft, J., Miller, J., and Mortha, A. (2013). The dendritic cell lineage: ontogeny and function of dendritic cells and their subsets in the steady state and the inflamed setting. *Annu Rev Immunol* 31, 563–604. doi: 10.1146/annurev-immunol-020711-074950

Mildner, A., Marinkovic, G., and Jung, S. (2016). Murine Monocytes: Origins, Subsets, Fates, and Functions. *Microbiol Spectr* 4. doi: 10.1128/microbiolspec.MCHD-0033-2016

Naik, S. H., Sathe, P., Park, H.-Y., Metcalf, D., Proietto, A. I., Dakic, A., et al. (2007). Development of plasmacytoid and conventional dendritic cell subtypes from single precursor cells derived in vitro and in vivo. *Nat Immunol* 8, 1217–1226. doi: 10.1038/ni1522

Ramírez-Toloza, G., and Ferreira, A. (2017). Trypanosoma cruzi Evades the Complement System as an Efficient Strategy to Survive in the Mammalian Host: The Specific Roles of Host/Parasite Molecules and Trypanosoma cruzi Calreticulin. *Front Microbiol* 8, 1667. doi: 10.3389/fmicb.2017.01667

Randolph, G. J., Jakubzick, C., and Qu, C. (2008). Antigen presentation by monocytes and monocyte-derived cells. *Curr Opin Immunol* 20, 52–60. doi: 10.1016/j.coi.2007.10.010

Romano, P. S., Cueto, J. A., Casassa, A. F., Vanrell, M. C., Gottlieb, R. A., and Colombo, M. I. (2012). Molecular and cellular mechanisms involved in the Trypanosoma cruzi/host cell interplay. *IUBMB Life* 64, 387–396. doi: 10.1002/iub.1019

Satpathy, A. T., Wu, X., Albring, J. C., and Murphy, K. M. (2012). Re(de)fining the dendritic cell lineage. *Nat Immunol* 13, 1145–1154. doi: 10.1038/ni.2467

Vu Manh, T.-P., Bertho, N., Hosmalin, A., Schwartz-Cornil, I., and Dalod, M. (2015). Investigating Evolutionary Conservation of Dendritic Cell Subset Identity and Functions. *Front Immunol* 6, 260. doi: 10.3389/fimmu.2015.00260

Zhou, J., Nefedova, Y., Lei, A., and Gabrilovich, D. (2018). Neutrophils and PMN-MDSC: Their biological role and interaction with stromal cells. *Semin Immunol* 35, 19–28. doi: 10.1016/j.smim.2017.12.004
